# A Novel AT-Rich DNA Recognition Mechanism for Bacterial Xenogeneic Silencer MvaT

**DOI:** 10.1371/journal.ppat.1004967

**Published:** 2015-06-11

**Authors:** Pengfei Ding, Kirsty A. McFarland, Shujuan Jin, Grace Tong, Bo Duan, Ally Yang, Timothy R. Hughes, Jun Liu, Simon L. Dove, William Wiley Navarre, Bin Xia

**Affiliations:** 1 Beijing Nuclear Magnetic Resonance Center, School of Life Sciences, College of Chemistry and Molecular Engineering, Peking University, Beijing, China; 2 Division of Infectious Diseases, Boston Children's Hospital, Harvard Medical School, Boston, Massachusetts, United States of America; 3 Department of Molecular Genetics, University of Toronto, Toronto, Ontario, Canada; 4 Donnelly Centre for Cellular and Biomolecular Research, University of Toronto, Toronto, Ontario, Canada; Purdue University, UNITED STATES

## Abstract

Bacterial xenogeneic silencing proteins selectively bind to and silence expression from many AT rich regions of the chromosome. They serve as master regulators of horizontally acquired DNA, including a large number of virulence genes. To date, three distinct families of xenogeneic silencers have been identified: H-NS of Proteobacteria, Lsr2 of the Actinomycetes, and MvaT of *Pseudomonas* sp. Although H-NS and Lsr2 family proteins are structurally different, they all recognize the AT-rich DNA minor groove through a common AT-hook-like motif, which is absent in the MvaT family. Thus, the DNA binding mechanism of MvaT has not been determined. Here, we report the characteristics of DNA sequences targeted by MvaT with protein binding microarrays, which indicates that MvaT prefers binding flexible DNA sequences with multiple TpA steps. We demonstrate that there are clear differences in sequence preferences between MvaT and the other two xenogeneic silencer families. We also determined the structure of the DNA-binding domain of MvaT in complex with a high affinity DNA dodecamer using solution NMR. This is the first experimental structure of a xenogeneic silencer in complex with DNA, which reveals that MvaT recognizes the AT-rich DNA both through base readout by an “AT-pincer” motif inserted into the minor groove and through shape readout by multiple lysine side chains interacting with the DNA sugar-phosphate backbone. Mutations of key MvaT residues for DNA binding confirm their importance with both *in vitro* and *in vivo* assays. This novel DNA binding mode enables MvaT to better tolerate GC-base pair interruptions in the binding site and less prefer A tract DNA when compared to H-NS and Lsr2. Comparison of MvaT with other bacterial xenogeneic silencers provides a clear picture that nature has evolved unique solutions for different bacterial genera to distinguish foreign from self DNA.

## Author Summary

During evolution, the bacteria frequently acquire new genes through horizontal transfer, in order to adapt new environments. However, foreign DNA sequences acquired are more likely to decrease rather than increase the fitness of the recipient bacteria. Therefore, many bacterial genera have evolved unique proteins to selectively repress the transcription of foreign genes. The opportunistic pathogen *Pseudomonas aeruginosa* is the principal cause of the morbidity and mortality in cystic fibrosis patients and is among the major causes of nosocomial infections. As a xenogeneic silencer, the MvaT protein of *P*. *aeruginosa* is a master regulator of horizontally acquired genes including many critical for drug resistance and virulence. Here, we have characterized the DNA sequences preferentially targeted by MvaT, and identified differences in sequence preferences between MvaT and other xenogeneic silencers. The high resolution structure of the DNA-binding domain of MvaT in complex with a high affinity DNA target reveals a novel AT-rich DNA minor groove recognition mechanism, which perfectly explains the characteristic of MvaT’s DNA sequence preferences. Comparison between MvaT and other bacterial xenogeneic silencers demonstrates how unique solutions have been employed by different bacterial genera in distinguishing foreign DNA from DNA of their own genome.

## Introduction

Horizontal gene transfer, or lateral gene transfer, refers to the acquisition of foreign genes not from a direct ancestor by an organism. It is a major evolutionary force in bacteria and unicellular eukaryotes [1], and a recent study even suggests that horizontal gene transfer may also contribute to animal evolution [2]. Although the bacteria frequently acquire foreign genes in order to adapt the environment, the expression of foreign genes in a new host is more likely to reduce the bacterial fitness [3]. Bacterial xenogeneic silencing proteins selectively bind to and repress the transcription from regions of DNA that are significantly more AT-rich than the overall genome, which are likely to be acquired through horizontal gene transfer [4–6]. By dampening the expression of many AT-rich genes, these silencers improve the probability that newly-acquired sequences will be tolerated by the recipient organism. A recent study also shows that one of the major functions of xenogeneic silencers is to suppress the toxic intragenic transcription initiation of horizontally acquired AT-rich DNA [7]. Silencing of foreign DNA can potentiate bacterial evolution by allowing a pool of potentially useful genes to exist cryptically in the population. Such genes occasionally find use when an individual cell in the population evolves the necessary regulatory circuitry to control the gene’s expression under the appropriate environmental and temporal contexts [8–13]. As a result of their activity, xenogeneic silencers are the master regulators of horizontally acquired sequences, including many critical for drug resistance and virulence, in a large number of important bacterial pathogens including *Mycobacteria*, *Vibrio*, *Salmonella*, *Escherichia*, *Yersinia*, *Bordetella*, and *Pseudomonas* [14–28].

Xenogeneic silencing proteins can be divided into three distinct groups based on sequence similarity: the H-NS-like proteins that are found in many genera of alpha, beta, and gamma-Proteobacteria, the Lsr2 proteins found in the actinobacteria, and the MvaT-like proteins of the pseudomonads [5]. They all share the same domain arrangement, with an N-terminal oligomerization domain and a C-terminal DNA binding domain [29, 30]. These proteins can cooperatively bind to large sections of DNA, and recent studies suggest that the formation of nucleoprotein filaments is essential for their gene-silencing function [31–34]. The DNA-binding domains of Lsr2 from *Mycobacterium tuberculosis* and H-NS from *Escherichia coli* and *Salmonella typhimurium* have been solved and their interactions with DNA have been modeled using titration data from NMR studies [16, 29, 35–37]. Interestingly, Lsr2 and H-NS, despite being structurally distinct, employ a common mechanism to selectively target AT-rich DNA by inserting an AT-hook-like “Q/RGR” motif into the minor groove of DNA. Another H-NS family protein Ler, with the AT-hook-like motif replaced by a “VGR” sequence, only inserts the arginine side chain into the minor groove of DNA and can alleviate H-NS mediated silencing of virulence operons in enteropathogeic *E*. *coli* [38].

MvaT was originally identified as a global regulator of virulence gene expression in *Pseudomonas aeruginosa* and subsequently shown to be a repressor of the *cupA* fimbrial cluster necessary for biofilm formation [23, 39, 40]. MvaT was characterized as an H-NS functional analog due to its ability to complement various phenotypes of the *E*. *coli Δhns* mutant [41]. Transcriptome and chromatin immunoprecipitation coupled with DNA microarrays (ChIP-on-chip) analysis have shown that MvaT, and its paralog MvaU, both preferentially bind AT-rich regions of the *P*. *aeruginosa* genome to regulate ~350 genes and that there is nearly complete overlap in the sets of genes under the control of each protein, although why apparently redundant paralogs exist is unclear [22, 42]. In addition to the *cupA* operon, a lot of virulence genes from *P*. *aeruginosa* are repressed by MvaT/MvaU, such as the genes required for synthesis of the redox-active pigment and toxin pyocyanin, and the genes related to the type III and type VI secretion systems [22]. Loss of both MvaT and MvaU is lethal to *P*. *aeruginosa* strain PAO1, a phenomenon that has been traced to the fact that these proteins inhibit activation of the Pf4 prophage [43]. Structural predictions suggest that MvaT/MvaU likely evolved from the H-NS family, however both proteins share very low sequence identity with H-NS and lack the canonical H-NS motif that contains the AT-hook-like structure [5, 41]. This leaves open the question of how the MvaT-like proteins target AT-rich DNA.

In this work we performed structural and functional analyses of the DNA binding properties of MvaT. High-throughput DNA binding assays indicate that MvaT prefers highly flexible sequences that contain multiple TpA steps and has considerable tolerance to GC-base pair interruptions in the binding sites. We solved the solution structure of the DNA-binding domain of MvaT in its free form and in complex with a high affinity DNA dodecamer and find that, like H-NS and Lsr2, MvaT recognizes structural features in the minor groove unique to AT-rich DNA. With an “AT-pincer” motif inserted into the minor groove and several lysine residues interacting with DNA sugar-phosphate backbone, the double helix was distorted by significantly opening of the minor groove, reminiscent of several other proteins that target flexible TpA-rich sequences. The roles of residues predicted to be critical for binding were assessed both *in vitro* and *in vivo* which reveal that, while single amino acid substitutions did not completely abolish DNA binding activity due to extensive contacts between MvaT and DNA, those substitutions with significantly reduced DNA binding activity render the protein non-functional *in vivo*. Finally, our structural data indicates that the MvaT-like proteins likely evolved from an H-NS paralog but acquired a mode of binding that is significantly distinct from other xenogeneic silencers.

## Results

### MvaT binds to its target DNA with slow off rate

Previous ChIP-on-chip analysis indicated that MvaT preferentially binds to AT-rich regions of the chromosome [22]. Electrophoretic mobility shift assays (EMSA) were performed with purified full-length MvaT against a radiolabeled 340 bp fragment of the *cupA1* promoter (%GC = 54), previously shown to bind MvaT *in vivo* and *in vitro*. For a negative control we employed a 204 bp fragment of the *P*. *aeruginosa* PAO1 gene *PA3900* (%GC = 74), which lies within a ~180 kb GC-rich region of the genome that does not contain any MvaT binding sites *in vivo*. MvaT was able to shift both labeled fragments of DNA when incubated with either fragment alone, with apparent dissociation constants of 57 ± 22 nM and 154 ± 53 nM towards the *cupA1* promoter and *PA3900* fragments, respectively. The binding affinity of MvaT for the GC-rich *PA3900* fragment is very close to that (~148 nM) determined previously with EMSA [32].

**Fig 1:**
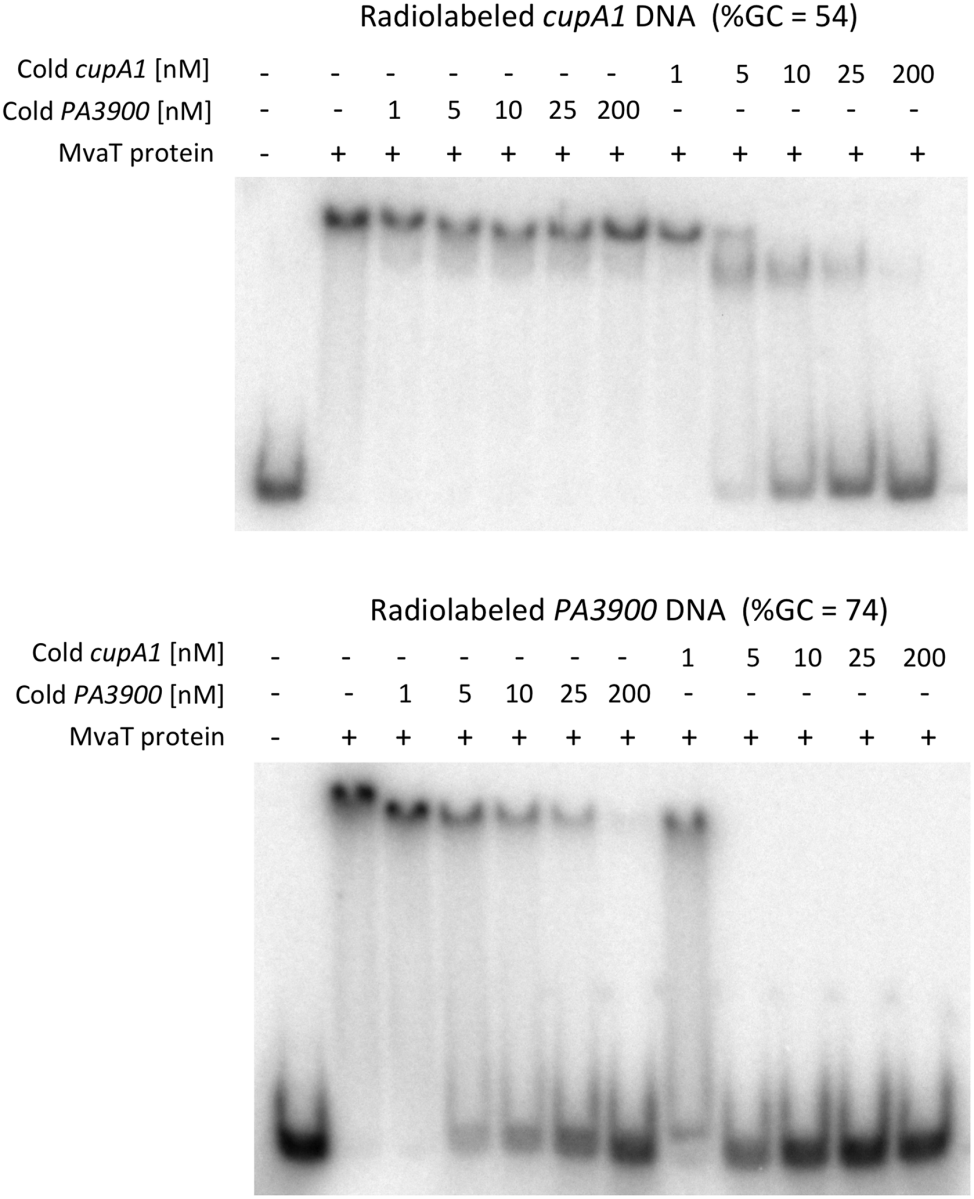
DNA competition binding assay for MvaT binding to *cupA1* promoter DNA and *PA3900* fragment. Complexes of MvaT (300 nM) with 1 nM radiolabeled AT-rich *cupA1* promoter DNA fragment (upper panel) or a radiolabeled GC-rich *PA3900* DNA fragment (lower panel), were pre-formed prior to the addition of excess unlabeled competitor DNA as indicated.

Although the affinity of MvaT towards the *cupA1* promoter DNA is only ~3 times higher than that of the *PA3900* fragment, significant differences were observed when competition assays were employed (Fig 1). MvaT, preincubated with the radiolabeled GC-rich *PA3900* fragment, was displaced by unlabeled *cupA1* promoter DNA at less than a 5-fold molar excess. In contrast, a 200-fold excess of unlabeled *PA3900* DNA was unable to displace MvaT from a pre-formed complex with the radiolabeled *cupA1* fragment. These data indicate that MvaT oligomers likely interact with GC-rich DNA through a series of coupled low affinity interactions. Subunits within MvaT oligomers dissociate from the GC-rich target frequently, enabling the protein oligomer to be removed from the DNA rapidly. On the *cupA1* promoter fragment, which is comparatively AT-rich, the MvaT protein forms an oligomeric complex sufficiently stable that a 25-fold excess of unlabeled *cupA1* DNA is required to displace it. This indicates that once bound to an appropriate target fragment, the MvaT oligomers make highly stable complexes that dissociate with very slow off rates. These results are consistent with the finding that MvaT can cooperatively bind to large sections of DNA and form nucleoprotein filaments [32]. In such nucleoprotein filaments, the dissociation of MvaT primarily occurs at the two ends where the energy cost is lower than inside the filament, resulting in slow off rates for the binding.

### MvaT exhibits subtle but clear differences in DNA sequence preferences from H-NS and Lsr2

The DNA binding specificity of MvaT was further interrogated using protein binding microarrays (PBM). GST-tagged MvaT was applied to microarrays containing 41,944 double-stranded 60-mer oligonucleotide target sequences each comprising a constant 25-mer primer sequence followed by a variable 35-mer sequence. Two different arrays (designated ME and HK) were designed such that all possible non-palindromic 8-mers are represented 32 times (16 times for palindromic 8-mers) on each array [44, 45]. The combined data (average) from the two independent array experiments were used to analyze the relative binding preferences, providing an unbiased estimate of relative preference to each 8-mer that is robust to variations in position, location and flanking sequence [29, 44].

The relative binding preference of MvaT for each 8-mer sequence was calculated using a rank-based, non-parametric statistical measure (*E*-score) that is largely invariant to protein concentrations. This facilitates a comparison of different experiments on the same scale, which is useful when assessing differences in binding targets between different proteins like MvaT and H-NS [44, 46]. The *E*-score ranges from 0.5 (highest) to -0.5 (lowest). Random permutations of the array data indicate there should be no random 8-mer sequence that achieves an *E*-score above 0.45 [47]. The PBM experiments identified multiple 8-mers with *E*-scores above 0.45 for MvaT and the highest score was around 0.49, indicating that some sequences were clearly preferential targets for binding by MvaT than others (S1 Dataset).

**Fig 2:**
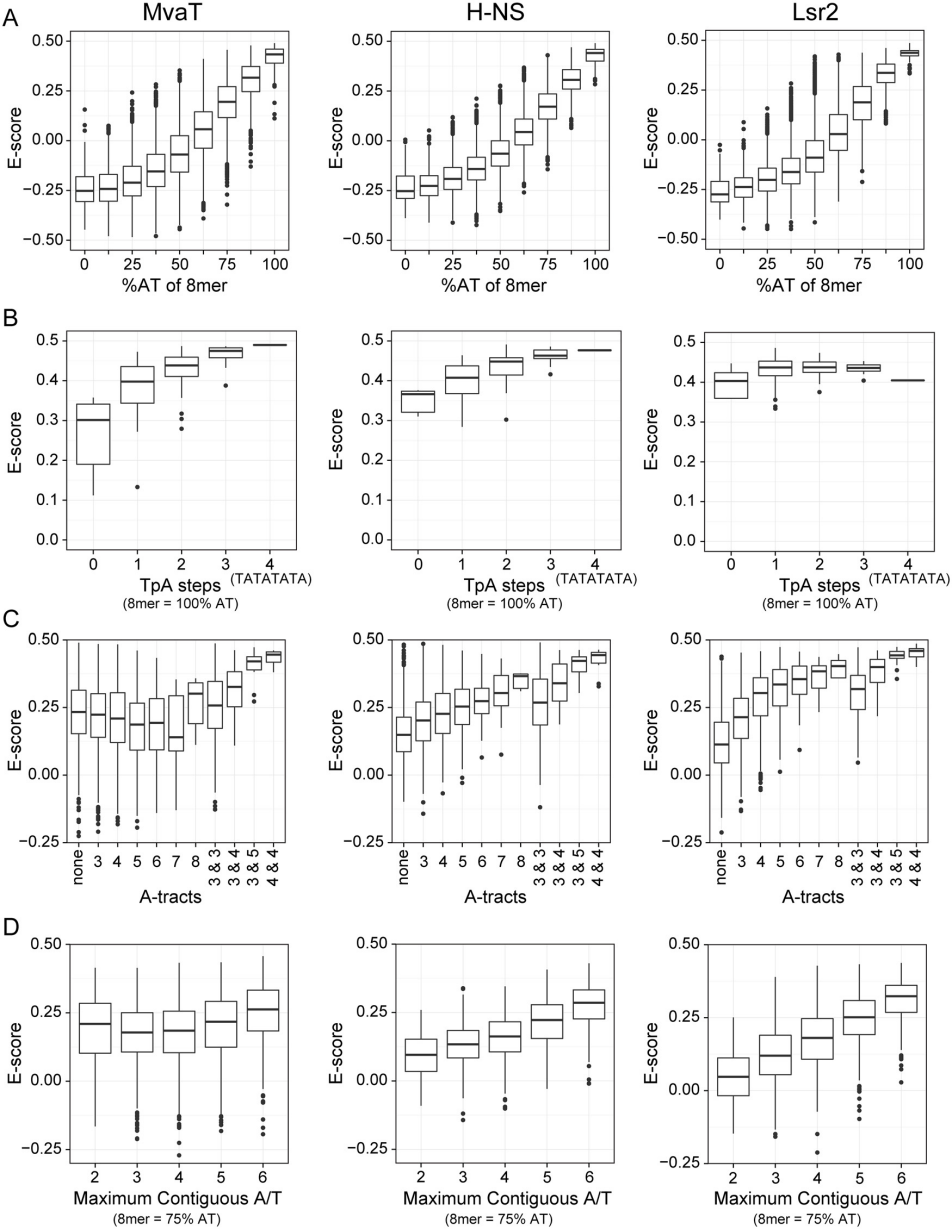
Protein binding microarray analysis of DNA binding preferences for MvaT, H-NS and Lsr2. PBM data for H-NS and Lsr2 was previously published [29]. Tukey boxplots show the *E*-score distributions of the 8-mer oligonucleotide sequences and the whiskers indicate points within the 1.5 inter-quartile range. In all charts the sequence parameters are plotted against the *E*-score, which reflects relative affinity for a particular sequence. (A) *E*-score vs. AT-content of 8-mer target sequence. (B) Number of TpA steps for oligos containing only A or T (100% AT). (C) Length of A-tract sequence(s) in 8-mers with %GC ≥ 75. Some sequences contain two A-tracts (e.g. AAAGGAAA) and in those cases the length of each is indicated. (D) *E*-score vs. maximum length of uninterrupted A or T bases in a 75% AT sequence. Sequences such as GCAATAAT have 6 adjacent A or T bases while the longest stretch of adjacent A or T bases in the 8-mer AAGTTCAA is 2.

As shown in Fig 2, the target preferences of MvaT were compared to those previously determined in similar experiments for both H-NS and Lsr2 [29]. Consistent with earlier studies, the sequences most tightly bound by all three proteins were overwhelmingly AT-rich, as the PBM *E*-score of MvaT is also positively correlated with the AT-content of 8-mers (Fig 2A). This provides the basis for their functional similarity and that MvaT can complement various phenotypes of the *E*. *coli Δhns* mutant [41].

However, a more detailed comparison of binding preferences between MvaT, H-NS, and Lsr2 indicates that subtle but important differences exist in their modes of binding. Both H-NS and MvaT are biased toward sequences containing multiple TpA dinucleotide steps (also called TpA steps), while TpA steps do not affect Lsr2 binding (Fig 2B). Indeed sequences with the highest affinities for both H-NS and MvaT were composed of multiple adjacent TpA steps and one of the highest scoring 8-mers for each was TATATATA. The absence of TpA steps from a given sequence, however, imparts a higher penalty on binding of MvaT than it does on either H-NS or Lsr2.

More than any other dinucleotide step including those containing a G or C, TpA steps confer the greatest amount of flexibility on DNA [48]. In stark contrast, A-tracts (sequences composed of several consecutive A or T bases without a TpA-step), are the most rigid and inflexible of all DNA sequences [49]. Base stacking within A-tract sequences also compresses the minor groove more than any other type of sequence. While the presence of A-tracts in a sequence generally improved binding by both H-NS and Lsr2, A-tracts imparted a significant penalty on binding by MvaT (Fig 2C).

The effect that G or C bases within the target site have on binding by the various proteins was also assessed (Fig 2D). Notably all proteins had lower affinity for AT-rich sequences when a single G or C nucleotide was placed in the center but the effect on binding by MvaT was the lowest. Binding of the proteins to 8-mer sequences containing six A or T bases and two G or C bases at various positions was assessed. 8-mer sequences with two G or C bases can have as many as 6 adjacent A or T nucleotides (e.g. AATATAGG), to as few as two (e.g. AAGTTGAA). These data indicate that the penalty for both H-NS and Lsr2 increases as the number of contiguous A or T nucleotides decreases. MvaT, on the other hand, seemed to be more tolerant to interruptions by G or C nucleotides within an AT-rich binding site and many high-scoring 8-mer sequences contained only sets of two adjacent T or A nucleotides. These differences in sequence preferences imply that the DNA recognition mechanism of MvaT may be different from that of H-NS and Lsr2.

Significant advances have recently been made in the computational prediction of specific DNA structure parameters. Structures of MvaT-bound sequences predicted by DNAshape [50] reveal that the top 20 scoring 100% AT 8-mer sequences are markedly different in structure from the 20 100% AT 8-mers with the lowest score (S1 Fig). Sequences with high MvaT binding *E*-scores all contain significant stretches where the minor groove exceeds 5.5 Å in width while the sequences that scored the lowest were predicted to contain much narrower minor grooves, typically below 4 Å. Top scoring sequences also had higher values for roll and propeller twist, and lower values for helical twist than the lower scoring sequences.

### MvaT C-terminal domain binds AT-rich DNA at the minor groove

**Fig 3:**
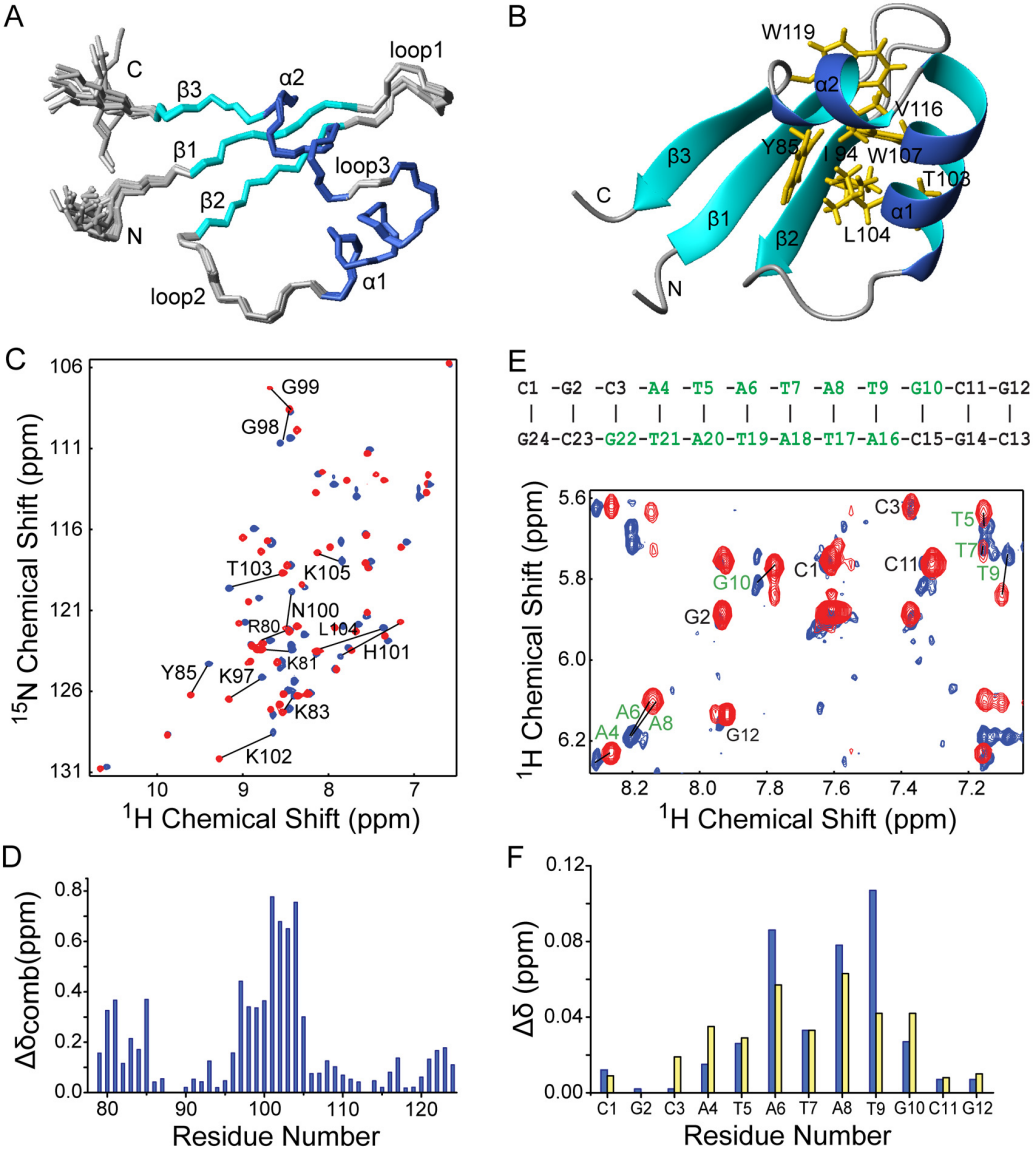
Solution structure of MvaT_ctd_ and its interaction with AT-rich DNA. (A) Superimposition of backbone traces for the ensemble of 20 structures of MvaT_ctd_. (B) Ribbon representation of the MvaT_ctd_ mean structure. The side chains of residues forming the hydrophobic core are shown in orange. (C) Overlay of 2D ^1^H-^15^N HSQC spectra of free (blue) and DNA-bound MvaT_ctd_ (red, concentration ratio 1:1). Residues showing significant chemical shift changes are indicated. (D) Combined ^1^H and ^15^N chemical shift differences (Δδ_comb_ = [δ_HN_^2^ + (δ_N_/6.5)^2^]^1/2^) between free and DNA bound-MavT_ctd_ are plotted against residue number (blue bars). (E) Overlay of the finger print region showing intraresidue H1’-H6/H8 NOE peaks of 2D ^1^H NOESY spectra of free (blue) and MvaT_ctd_-bound DNA (red; concentration ratio 1:1). The sequence and secondary structure of the DNA dodecamer is shown above, with residues affected by MvaT_ctd_ binding indicated in green. (F) ^1^H chemical shift differences (Δδ) for H1’ (blue bars) and H6/H8 (yellow bars) chemical shifts between free and MvaT_ctd_-bound DNA are plotted against residue number.

We determined the solution structure of the C-terminal DNA-binding domain of MvaT (MvaT_ctd_, residues 77–124) from *P*. *aeruginosa* strain PAO1 using nuclear magnetic resonance (NMR), with nearly complete resonance assignments achieved (S2 Fig). Restraints and structural statistics are summarized in Table 1. MvaT_ctd_ consists of a three-stranded antiparallel β–sheet (β1, residues 83–86; β2, residues 93–96; β3, residues 120–122), and two α-helices (α1, residues 102–111; α2, residues 113–119). MvaT_ctd_ adopts the topology of β1-β2-α1-α2-β3, and a loop (loop2, residues 97–101) links the β1-β2 hairpin and the helices α1-α2. The N- and C-terminal regions are highly flexible (Fig 3A). The two consecutive helices are packed on one side of the 3-stranded β–sheet and form a hydrophobic core composed of residues Tyr85, Ile94, Thr96, Thr103, Leu104, Trp107, Trp111, Val116, Trp119 and Ala120 (Fig 3B). The overall fold and secondary structure composition of MvaT_ctd_ are similar to those of the C-terminal domain of H-NS (H-NS_ctd_), except that H-NS_ctd_ does not have a third strand β3 and it is a 3_10_ helix instead of the helix α2 in MvaT_ctd_ [29].

**Table 1:** Restraints and structural statistics for MvaT_ctd_ and its complex with DNA.

	MvaT_ctd_	MvaT_ctd_/DNA
**Restraints for protein**		
Total NOE	1820	2573
Intra-residue	557	662
Inter-residue	718	1200
Sequential (|*i*—*j*| = 1)	299	460
Nonsequential (|*i*—*j*| > 1)	419	740
Ambiguous	545	711
Hydrogen bonds	16	16
Dihedral angle restraints	64	70
Φ Angle	32	35
Ψ Angle	32	35
Chirality restraints	170	170
ω Angle	46	46
Side chain	124	124
**Restraints for DNA**		
Total NOE		911
Intra-nucleoside		521
Inter-nucleoside		390
Sequential (|*i*—*j*| = 1)		333
Nonsequential (|*i*—*j*| > 1)		57
Hydrogen bonds		30
Sugar pucker		24
Backbone dihedral angle		158
**Protein–DNA intermolecular**		57
**Restraints violations**		
Distance restraints (> 0.2 Å)	0	2
Dihedral angle restraints (> 5°)	0	0
**Average pairwise RMSD** * **(Å)**		
Protein (all heavy atom)	0.95 ± 0.19	0.58 ± 0.10
Protein (backbone heavy atom)	0.52 ± 0.15	0.17 ± 0.06
DNA (all heavy atom)		0.31 ± 0.10
Protein and DNA (all heavy atom)		0.49 ± 0.08
**Ramachadran plot (%)**		
Most favored regions	85.7	88.9
Additionally allowed regions	14.3	11.1
Generously allowed regions	0	0
Disallowed regions	0	0

*Pairwise RMSD was calculated among 20 refined structures over residues A79-G124 (MvaT_ctd_) and/or C1-G24 (DNA).

The interaction surfaces of MvaT_ctd_ and DNA were mapped by NMR titration experiments. A DNA duplex d(CGCATATATGCG)_2_, which we refer to as “3AT”, was chosen as it contains a sequence among those with highest scores from our PBM study. Comparison of 2D ^1^H-^15^N HSQC spectra of MvaT_ctd_ in free and DNA-bound form reveals that the residues with significant combined NH chemical shift differences (Δδ_comb_ > 0.25 ppm) are Arg80, Lys81, Tyr85, and Lys97-Lys105 (Fig 3C and 3D), mainly located on the N-terminal region, loop2 and the beginning of helix α1. These residues, except Tyr85, are clustered on the structure of MvaT_ctd_, constituting the DNA binding site. Tyr85 is probably affected indirectly through its aromatic side chain, which protrudes straight towards loop2. The stoichiometry of binding is one molecule of MvaT_ctd_ to one molecule of 3AT duplex as assessed by fitting of the chemical shift changes with various DNA concentrations, which was also confirmed by the results of isothermal titration calorimetry (ITC, see below). By comparing 2D ^1^H NOESY spectra of the DNA, free or in complex with protein, we also mapped the regions of 3AT that interact with MvaT_ctd_. Significant intra-residual H1’-H6/H8 NOE peak shifts (H1’ or H6/H8 Δδ > 0.025 ppm) occur at the central ATATATG residues (Fig 3E and 3F).

To confirm that MvaT_ctd_ binds the minor groove we performed a competition experiment using netropsin, a natural oligopeptide that binds the minor groove of AT-rich DNA. With the addition of increasing concentration of netropsin into a sample of ^15^N-MvaT_ctd_ containing a two-fold excess of 3AT, the NMR signals of MvaT_ctd_ shifted from the DNA-bound form back to the free form gradually, indicating that the MvaT_ctd_/DNA complex was disrupted by netropsin in a concentration-dependent manner. At a netropsin/DNA ratio of 2.5:1, the 2D ^1^H-^15^N HSQC spectrum is nearly identical to that of free MvaT_ctd_, indicating that netropsin almost completely releases MvaT_ctd_ from 3AT.

### Structure of the MvaT_ctd_/DNA complex reveals a novel DNA binding mechanism

**Fig 4:**
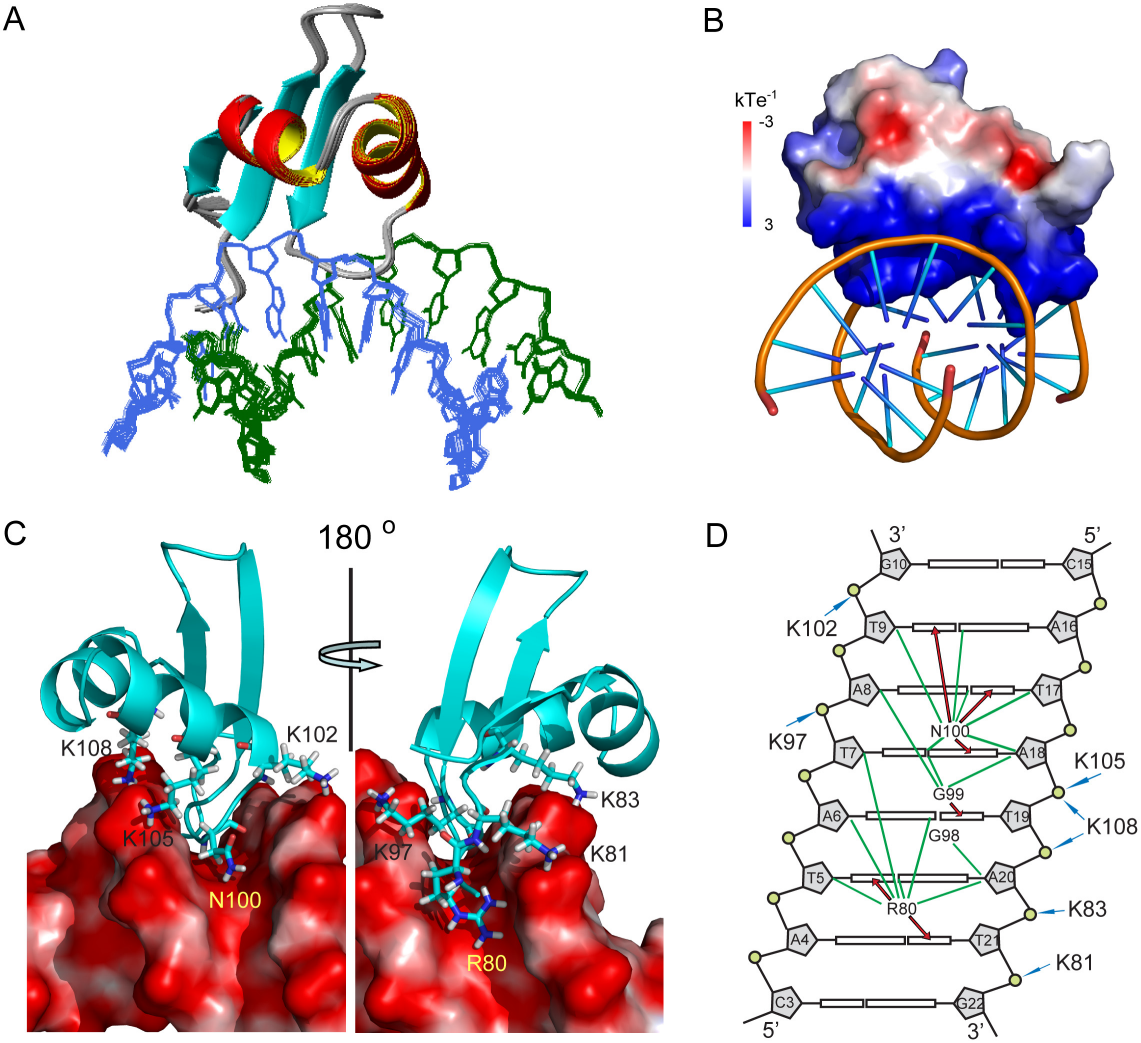
Structure of MvaT_ctd_ and AT-rich DNA complex. (A) Superimposition for the ensemble of 20 structures of MvaT_ctd_/DNA complex. Protein backbone is represented by ribbon and the target DNA is shown as lines. (B) Electrostatic potential of MvaT_ctd_, calculated with APBS in the absence of DNA. (C) Close view of the binding interface. Residues of MvaT_ctd_ involved in DNA recognition are shown as sticks with one-letter amino acid code and residue number labeled. (D) Schematic representation of the intermolecular contacts. The DNA is drawn as a cylindrical projection viewed from the minor groove side. Hydrogen bonds between MvaT_ctd_ and DNA bases are indicated by red arrows. Green lines show the hydrophobic contacts. The interactions between positively charged lysine residues and DNA backbone phosphate groups are indicated by blue arrows.

Unlike Lsr2 and H-NS [16, 29], the DNA-bound MvaT_ctd_ has a single set of NH signals, and none of the NH signals disappear upon DNA binding (S2 Fig), enabling us to determine the solution structure of MvaT_ctd_ in complex with the 3AT dodecamer (Fig 4A). The MvaT_ctd_/3AT complex structure was determined using 3,541 experimental distance restraints, including 57 intermolecular distance restraints, and the backbone heavy atom RMSD of the 20 final structures with lowest AMBER energies is 0.49 Å (Table 1).

The structure of DNA-bound MvaT_ctd_ is almost the same as that of the free MvaT_ctd_, as their backbone heavy atom RMSD between the mean structures is only 0.90 Å. The major difference is that the N-terminal region becomes more rigid, consistent with the gradual emergence of the ε-NH signal of the Arg80 side chain when bound to 3AT (S2B Fig). The interaction surface of MvaT_ctd_ is positively charged (Fig 4B) and buries a surface area of 1,707±14 Å^2^. Loop2 of MvaT_ctd_ is partially inserted into the DNA minor groove with the backbone of residues Gly99 and Asn100 making contacts to the AT base pairs at the bottom, while residues Lys97 and Gly98 are tilted toward the top of the minor groove. The backbone amides of Gly99 and Asn100 form hydrogen bonds with the O2 atom of T19 and the N3 atom of A18, respectively. The side chain of Asn100 extends along the minor groove, and its δ-NH_2_ group forms hydrogen bonds with the O2 atoms of T9 and T17 (Fig 4C and 4D). In addition, the side chain of Arg80 is also inserted into the DNA minor groove with its guanidino group hydrogen bonded to O2 atoms of T5 and T21 (Fig 4C and 4D) and thus stabilizes the N-terminal region of MvaT_ctd_. The side chains of Arg80 and Asn100 are pointed away from each other, and occupy a region covering all six AT base pairs together with loop2. This DNA binding motif was thus given the name “AT-pincer” as the minor groove-intercalating residues are from two different loops of MvaT_ctd_. Besides interacting with the DNA minor groove, MvaT_ctd_ also contains six lysine residues (Lys81, Lys83, Lys97, Lys102, Lys105 and Lys108) that are well-positioned to make hydrophobic or electrostatic contacts with the DNA sugar-phosphate backbone through side chain methylene groups or the ε-amino groups, respectively (Fig 4C and 4D). The Lys81 side chain is stabilized upon binding 3AT, while the side chains of Lys83, Lys97, Lys102, Lys105 and Lys108 are already well-defined in the structure of free MvaT_ctd_, as these lysine side chains are stabilized by hydrophobic interactions with nearby residues. This extensive network of lysine residues significantly increases the DNA contact surface and is a distinguishing feature of MvaT_ctd_. This AT-rich DNA binding mode is clearly different from that of H-NS and Lsr2, which share a common DNA binding mechanism through the AT-hook-like motif (detailed comparison in the Discussion section).

**Fig 5:**
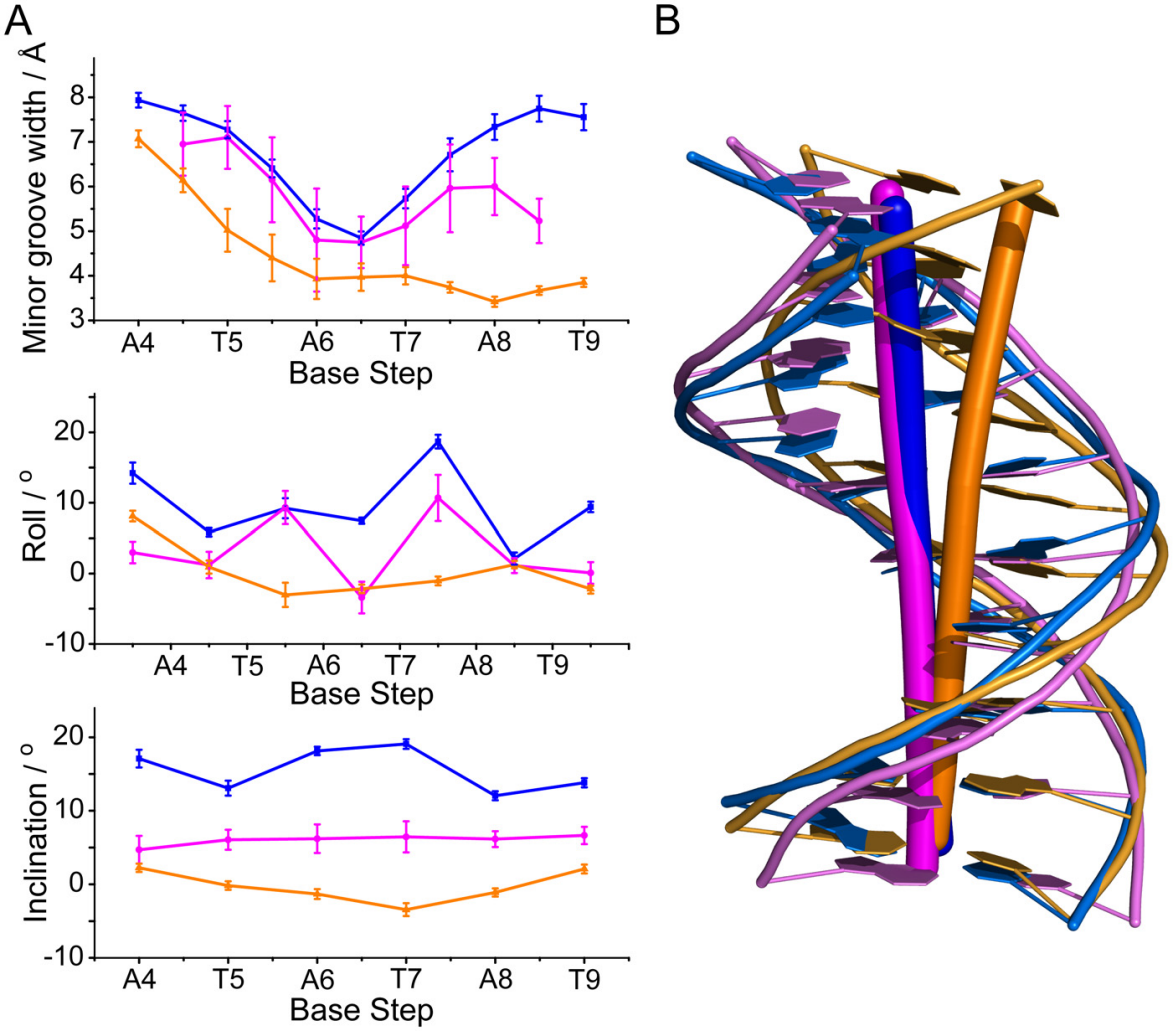
Analysis of MvaT_ctd_-bound DNA conformation. (A) Selected helix parameters of the solution structures of MvaT_ctd_-bound 3AT DNA (blue), a free DNA with ATATAT sequence (PDB 2LWG) (magenta) and an A-tract DNA with AAAAAA sequence (PDB 1FZX [52]) (gold). Average minor groove width, roll and inclination angles including standard deviations are shown. Base steps of 3AT are indicated. (B) Mean structures of MvaT_ctd_-bound 3AT (blue), 2LWG (magenta) and 1FZX (gold). Axes are shown by sticks.

Upon binding of MvaT_ctd_, the minor groove of 3AT is expanded and the base-stacking geometry is significantly rearranged. MvaT_ctd_-bound 3AT shows increased roll and inclination angles compared with a free DNA decamer d(GGATATATCC)_2_ with the same central ATATAT sequence (PDB 2LWG [51]), ~9.6° and ~15.5° per step in average respectively (Fig 5A), indicating that the base pairs locally bend toward the major groove and thus open the minor groove [49]. The minor groove width of MvaT_ctd_-bound 3AT reaches a minimum at the middle A_6_pT_7_ base step and progressively widens toward each end of the ATATAT sequence. The side chains of Arg80 and Asn100 are located at the A_4_pT_5_ and A_8_pT_9_ base steps respectively, where the minor groove widths are significantly widened compared with those in the structure of 2LWG. The above mentioned lysine side chains are fit well to the backbone of 3AT in the MvaT_ctd_/DNA complex, while their conformations remain similar to those in free protein. It is thus likely that the distortion of the DNA duplex is to accommodate the conformation of this “lysine network”. In contrast, A-tract DNA was reported to possess a very narrow minor groove, and the roll and inclination angles are consecutively small or negative [49, 52]. A-tract DNA also exhibits an overall bend of ~15° towards the minor groove [53], whereas the free and MvaT_ctd_-bound 3AT are nearly straight (Fig 5B). These may explain why A-tract DNA is not preferred by MvaT, as the rigid A-tract DNA does not have a favorable conformation for MvaT binding.

### Both “AT-pincer” and “lysine network” are important for DNA binding

To study the importance of the residues implicated by the MvaT_ctd_/DNA complex structure, we generated a series of MvaT_ctd_ mutants containing substitutions R80A, K81A, K83A, K97A, N100A, K102A, K105A and K108A. All these mutations except K83A do not have a significant effect on the overall structure of MvaT_ctd_ as the 2D ^1^H-^15^N HSQC spectra of these mutants are similar to that of wild-type (WT) MvaT_ctd_ (S3 Fig). Since the NH signals of K83A are completely different from that of WT MvaT_ctd_, the mutation K83A likely resulted in an overall fold change. This indicates Lys83 side chain plays an important role in stabilizing the structure, as it forms a salt bridge with Glu117 and its aliphatic groups make hydrophobic contact with Tyr85, Ala120, and Leu122.

Each mutant, excluding K83A, was subsequently titrated with the 3AT dodecamer and assessed by NMR (S4 Fig). For mutants K81A and K97A, the NH signals shift patterns during DNA titration are quite similar to those of WT MvaT_ctd_ in terms of scale and direction, suggesting that the two mutations should not have a significant effect on the DNA binding of MvaT_ctd_. Mutants R80A, K102A show reduced chemical shift changes, but with similar NH signals shift directions to those of WT MvaT_ctd_. Mutations N100A, K105A and K108A have the most profound effects on NH signal perturbation patterns induced by 3AT, with much smaller NH chemical shift changes and some NH signals shift directions are quite different from those of WT MvaT_ctd_, indicating that these mutations may significantly perturb the DNA binding mode of MvaT_ctd_.

**Fig 6:**
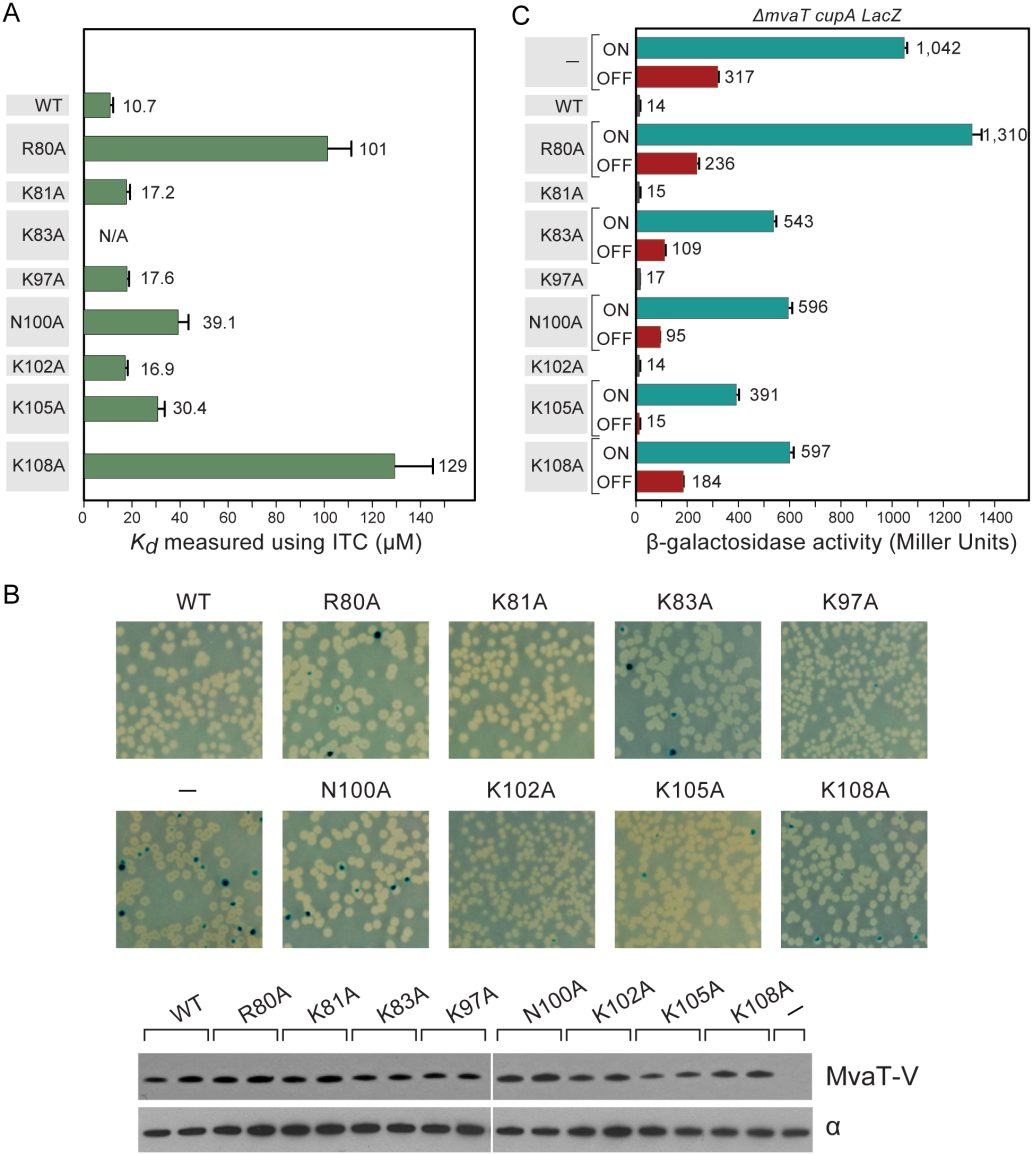
Characterization of MvaT mutants. (A) Dissociation constants for WT MvaT_ctd_ and its mutants when binding with 3AT as measured using ITC. Error bars represent standard deviation. (B) Phenotypes of *cupA lacZ* strains containing plasmids directing the expression of WT MvaT or its mutants (upper panel). The phase-variable expression of *cupA* genes can be repressed by providing the *mvaT* gene in trans, giving rise to white colonies. Derivative with functionally impaired MvaT gives rise to blue (phase-ON) and white (phase-OFF) colonies. Western blot analysis probed with antibody against VSV-G tag (lower panel). Sample loading control of western blot is probed with antibody against the α subunit of RNA polymerase. (C) Quantification of the *cupA lacZ* expression in cultures of strains containing WT MvaT or its mutants. Error bars represent standard deviation of the mean (n = 3).

ITC experiments were performed to characterize the binding affinities between DNA and MvaT_ctd_ or its mutants (Figs 6A and S5). As expected, WT MvaT_ctd_ exhibits the highest affinity for 3AT, with a *K*_*d*_ of about 10.7 μM. The binding affinities for mutants R80A and K108A are over 10 times weaker than WT MvaT_ctd_, while *K*_*d*_ values of mutants N100A and K105A are increased by over 3 times. On the contrary, mutations K81A, K97A and K102A only result in slightly lower DNA binding affinity (*K*_*d*_ increased by ~70%).

MvaT_ctd_ binds to a GC-rich DNA, d(CGCGCGCG)_2_ duplex, with much weaker affinity (*K*_*d*_ ~360 μM) (S5I Fig). It is noteworthy that WT MvaT_ctd_ and its mutants bind to AT-rich DNA in an endothermic manner, indicating that the binding is an entropy-driven process. It has been suggested that the large positive enthalpy change is due to the desolvation of the DNA minor groove and distortion of DNA duplex as we observed in the MvaT_ctd_/DNA complex [54]. The favorable entropy change can be attributed to the displacement of highly ordered water molecules in the DNA minor groove [55]. In contrast, the binding of MvaT_ctd_ to GC-rich DNA is driven by both enthalpy and entropy, suggesting that MvaT binds GC-rich DNA in quite different mode.

### MvaT mutants with significantly reduced DNA binding affinity are functionally impaired

We next tested whether those residues of MvaT that are implicated in DNA binding are important for the function of MvaT in cells of *P*. *aeruginosa*. It has been shown previously that MvaT in *P*. *aeruginosa* represses phase-variable expression of the *cupA* fimbrial genes [23]. In the strain PAO1 *ΔmvaT cupA1 lacZ*, where the *lacZ* gene is positioned downstream of the chromosomal *cupA1* gene, the *cupA* genes are expressed in a phase-variable manner, manifested by the appearance of blue and white colonies on LB agar plates that contain the chromogenic substrate 5-bromo-4-chloro-3-indolyl-β-D-galactopyranoside (X-Gal). This reversible ON–OFF switching of *cupA* gene expression observed in cells of the *ΔmvaT* mutant strain can be repressed by providing the *mvaT* gene in trans, giving rise to white colonies on LB agar plates containing X-Gal [23].

To test whether the MvaT mutants containing substitutions R80A, K81A, K83A, K97A, N100A, K102A, K105A and K108A could repress phase-variable expression of the *cupA* fimbrial genes as efficiently as WT MvaT, we introduced into cells of the reporter strain PAO1 *ΔmvaT cupA lacZ* plasmids directing the synthesis of either WT MvaT or an MvaT mutant, each containing a vesicular stomatitis virus-glycoprotein (VSV-G) epitope tag at its C-terminus. Cells were then grown on LB agar plates containing X-Gal. Western blotting using an antibody against the VSV-G epitope tag revealed that all proteins were made at comparable amounts except for K105A, which was slightly less abundant than the others (Fig 6B).

Unlike WT MvaT containing a VSV-G epitope tag (MvaT-V), the MvaT-V mutants R80A, K83A, N100A, K105A and K108A failed to repress phase-variable expression of the *cupA lacZ* reporter (Fig 6B). Conversely, MvaT-V mutants K81A, K97A, and K102A could repress *cupA* activity, similarly to WT MvaT-V. To further elucidate the effect of the MvaT-V variants on *cupA* repression, *lacZ* expression was quantified in cells grown in liquid culture by β-galactosidase assay. This confirmed that the MvaT-V variants K81A, K97A, and K102A could repress *cupA* activity just as well as WT MvaT-V (Fig 6C). Of the MvaT-V mutants that were unable to repress *cupA*, the R80A variant was most similar to the empty vector control and thus the most defective, whereas the K105A mutant was least defective. Because substitution K105A appears to reduce the abundance of MvaT-V, it is possible that the reduced ability of the K105A mutant to repress phase-variable expression of the *cupA* genes can in part be explained by the effects of this substitution on protein abundance. The impaired function for the K83A variant likely resulted from the change of protein fold as discussed above. Both mutants N100A and K108A were significantly impaired in their ability to repress *cupA* expression to similar extents (Fig 6C). These findings demonstrate that mutants of MvaT that exhibit notably reduced DNA binding affinity *in vitro* are therefore functionally impaired *in vivo*.

## Discussion

In this study, we have performed a comprehensive analysis of the DNA binding properties and mechanism of the xenogeneic silencer MvaT from the opportunistic pathogen *P*. *aeruginosa*. We have demonstrated that MvaT has higher binding affinity towards the AT-rich genomic target DNA, compared with the GC-rich non-target DNA. In addition, MvaT binds its target DNA with much slower off rate, which should also contribute to its selectivity for target DNA. The PBM data reveal that MvaT in general has similar DNA sequence preferences as H-NS and Lsr2. However, it is also found that MvaT prefers AT-rich sequences with multiple TpA steps and has considerable tolerance to GC-base pair interruptions, which is unique to MvaT. Our structural and functional evidence indicates that MvaT employs a novel AT-rich DNA recognition mechanism, distinct from that of H-NS and Lsr2, to carry out the xenogeneic silencing function.

Although the DNA binding affinity of a single DNA binding domain is relatively low (~10 μM), the full-length MvaT binds its genomic AT-rich target DNA with much higher affinity due to the multivalent effect, since the full-length MvaT is oligomerized in solution through its N-terminal domains [30], and thus it binds the target DNA containing multiple binding sites as a oligomer with multiple DNA binding domains. As a result, the binding energy of each full-length MvaT molecule in a nucleoprotein complex on DNA is not only contributed by its interaction with DNA, but also by its interaction with neighboring molecules of DNA-bound MvaT (mediated through their N-terminal domains), which significantly increase the overall stability of the complex. The multivalent interaction is widespread in chromatin biology [56], and it is also employed by other xenogeneic silencers. The *K*_*d*_ values of H-NS_ctd_ (~9 μM) and Lsr2_ctd_ (~4 μM) for binding 3AT DNA are similar to that of MvaT_ctd_ [29], while their full-length proteins also bind DNA with much higher affinities [33, 57]. Multivalent binding consisted of individual weak interaction is more susceptible to competition than a monovalent tight binding could be [58], which make it easier for counter silencers, such as Ler, to alleviate the repression of xenogeneic silencers in the regulation of gene expression [59].

**Fig 7:**
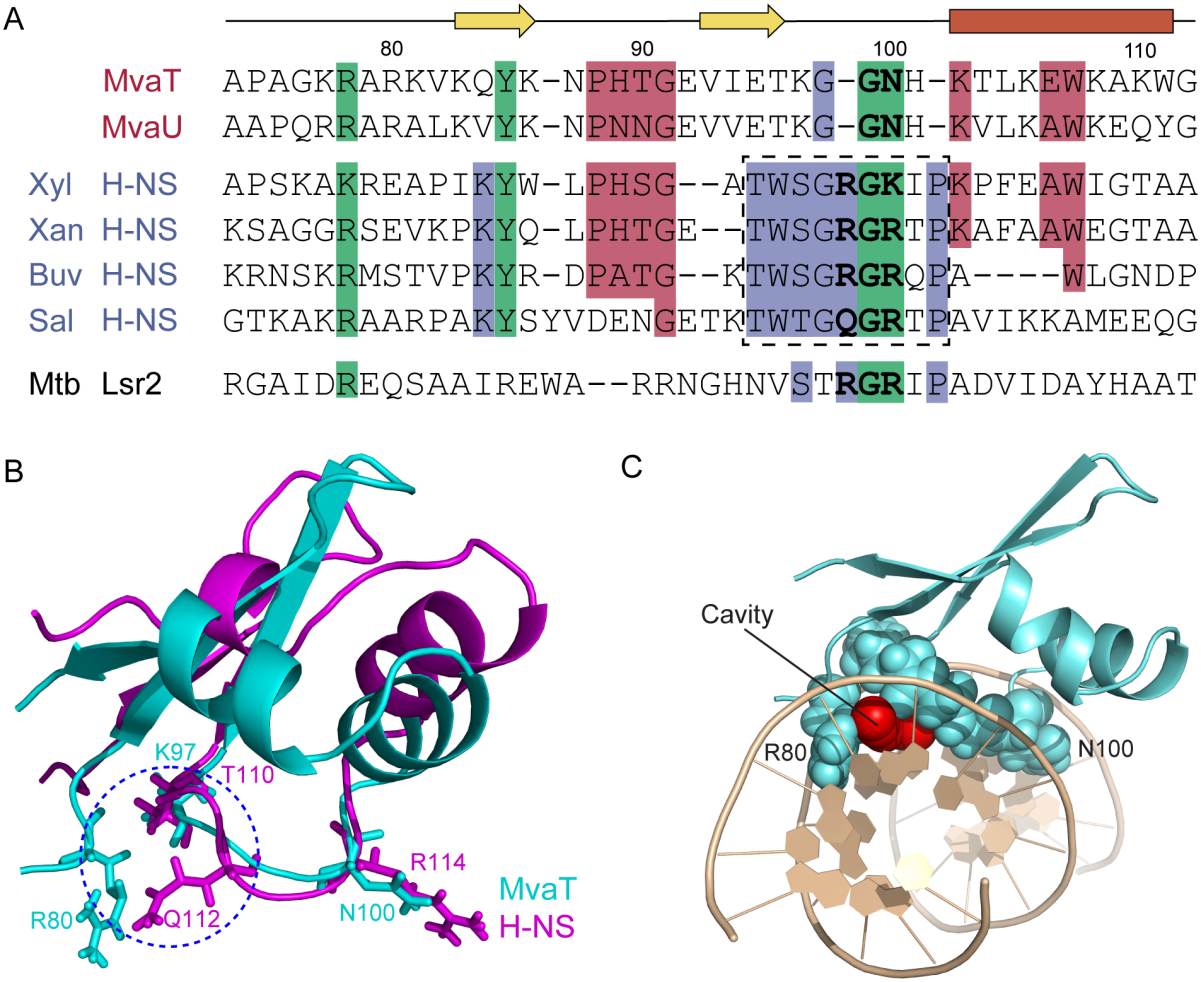
Sequence alignments and structural comparisons of MvaT_ctd_ with AT-hook-like motif containing DNA-binding domains. (A) Alignment of *Pseudomonas* MvaT with members of the H-NS family reveals regions of similarity outside of the canonical H-NS motif. Clustal Omega alignment of C-terminal domains of MvaT and MvaU from *P*. *aeruginosa* PAO1 with Bv3F from *Burkholderia vietnamiensis* G4 (Buv), H-NS from *S*. *typhimurium* (Sal), *Xanthomonas albilineans* (Xan), and *X*. *fastidiosa* (Xyl), and Lsr2 from *M*. *tuberculosis*. The canonical H-NS motif is boxed. In bold are the residues corresponding to the AT-hook-like motif in Lsr2 and H-NS. (B) Superimposing structures of MvaT_ctd_ (cyan) and *S*. *typhimurium* H-NS_ctd_ (magenta). Side chain conformations of K97 and N100 of MvaT_ctd_ are nearly identical to those of T110 and R114 of H-NS_ctd_, respectively. MvaT_ctd_ lacks the corresponding residue of Q112, functioning as the first critical residue of the “Q/RGR” AT-hook-like structure in H-NS_ctd_. When MvaT_ctd_ is complexed with DNA, side chain of R80 occupies similar position as the side chain of Q112 from H-NS_ctd_. A blue circle is drawn to indicate the gap between the R80 side chain and the backbone of loop2. (C) MvaT_ctd_/DNA complex structure viewed from one end of the double helix. The cavity above A6-T19 base pair is shown by red spheres.

The structure of MvaT_ctd_ in complex with 3AT DNA reveals that the DNA binding domain of MvaT recognizes AT-rich DNA through both the “AT-pincer” motif inserted into the DNA minor groove and the “lysine network” with multiple positive charges interacting with DNA backbone phosphates. MvaT lacks the AT-hook-like “Q/RGR” motif that is critical for both H-NS and Lsr2 to bind DNA, although they all specifically target the minor groove of AT-rich DNA. Homology predictions indicate that MvaT could share structural similarity to the H-NS family proteins found in the *Xanthomonadaceae* including the plant pathogen *Xylella fastidiosa*. An alignment of the MvaT DNA-binding domain with a diverse set of H-NS family members indicates that MvaT shares similarity to some H-NS homologs in regions immediately N-terminal to the canonical H-NS motif (TW(S/T)G(Q/R)GRTP). The canonical motif, however, is replaced by a different sequence (VIETKGGNH) that is highly conserved among the MvaT-like proteins and absent in members of the H-NS family (Fig 7A).

The overall fold of MvaT_ctd_ is similar to that of H-NS_ctd_, although the relative orientations of the helices and the β sheet for these two proteins are different (Fig 7B). Loop2 of MvaT_ctd_, comprising the sequence (KGGNH), is two residues shorter than the corresponding loop of H-NS_ctd_, where the AT-hook-like “Q/RGR” motif is embedded (Fig 7A and 7B). The first missing residue in MvaT corresponds to the minor groove binding “Q/R” of the “Q/RGR” motif. The AT-hook-like motifs of H-NS and Lsr2 are inserted into the DNA minor groove adopting a flat conformation with the side chains of the first “Q/R” and last “R” residues extended in opposite direction parallel to the minor groove floor. However, upon MvaT binding DNA, only Gly99 and Asn100 of loop2 insert into the minor groove and form hydrogen bonds with the bases. The side chain of Asn100 has a similar conformation as the last arginine of the “Q/RGR” motif, and thus the MvaT Gly99 and Asn100 residues appear to function as half of the AT-hook structure. Interestingly, the role of the missing first “Q/R” residue of the AT-hook-like motif is compensated by the side chain of Arg80 from the N-terminal region of MvaT_ctd_, which thus enables MvaT to recognize the AT-rich DNA minor groove with an “AT-pincer” motif composed of residues Arg80 and Gly99-Asn100. As a result, there is a cavity in the protein/DNA interface with a minimum radius of 1.2 Å above the A6-T19 base pair (Fig 7B and 7C) as calculated using CAVERS [60], which could theoretically accommodate an exocyclic NH_2_ group from a GC-base pair. This should explain the higher tolerance of MvaT for GC-base pair interruptions as revealed by our PBM data, since the DNA minor groove is almost fully occupied by the AT-hook-like motifs of H-NS and Lsr2.

It was recently proposed that both direct (base readout) and indirect (shape readout) DNA readouts are important in DNA recognitions for most DNA binding proteins [61, 62]. While the “AT-pincer” motif of MvaT inserts into the minor groove and achieves the base readout, the side chains of several lysine residues (residues 81, 83, 97, 102, 105 and 108) of MvaT interact with the DNA sugar-phosphate backbone and constitute the minor groove shape readout. Mutagenesis studies indicate that both the “AT-pincer” motif and the “lysine network” are important for the DNA binding affinity of MvaT_ctd_. Our functional assay also reveals that point mutation of residue Arg80 or Asn100 of the “AT pincer” motif, as well as residue Lys105 or Lys108 of the “lysine network”, impairs the function of MvaT to repress phase-variable expression of the *cupA lacZ* reporter. Structural comparison of free and DNA-bound MvaT_ctd_ shows that most of the key lysine side chains do not change their conformations upon binding DNA, while 3AT adopts a nearly straight conformation with its minor groove widened to accommodate MvaT binding. This can explain why MvaT favors flexible DNA sequences with multiple TpA steps over A-tract DNA. It is well known that A-tract DNA is more rigid and intrinsically bent ~15° towards the minor groove [49, 53], and therefore it would be energetically costly for the A-tract DNA to open up its minor groove and adopt a favorable conformation for MvaT binding.

The differences in DNA distortion upon binding by MvaT and H-NS/Lsr2 are notable. Minor groove distortion is most dramatically illustrated by the eukaryotic TATA binding protein (TBP), which opens the minor groove to unwind the double helix by approximately 120°, and compresses the major groove to bend the DNA by almost 80°. The critical parameter for TBP binding is not the sequence per se, but rather the inherent flexibility of the TpA step, which allows the protein to dramatically open the minor groove and make extensive contacts with both the phosphate backbone and bases that make up the floor of the groove. The distortions triggered in DNA when bound by MvaT are significant, although relatively small in comparison to TBP. At the other extreme are the eukaryotic HMG-I(Y) proteins that contain narrow and flexible AT-hook motifs, each composed of an R-G-R-P motif. The AT-hook forms a narrow crescent shaped structure that intercalates into the minor groove without significantly distorting the helix trajectory [63], which is probably the case of H-NS and Lsr2 when binding DNA with their AT-hook-like motifs. The MvaT DNA binding mode is least similar to Lsr2, which prefers A-tract DNA with a narrow minor groove and is largely insensitive to TpA steps.

Among the three families of bacterial xenogeneic silencers, H-NS and Lsr2 share a common AT-hook-like DNA recognition mechanism even though they are structurally dissimilar, while the sequence and structural similarities between MvaT and H-NS suggest they may share a common evolutionary origin. It is curious that MvaT adopts a distinct binding mode to recognize similar, but not identical, DNA sequence targets. Genomic analysis suggests that xenogeneic sequences frequently display higher AT-content compared to the host genome [64]. However, the mean genome-wide AT-contents for different bacterial genera could be quite different, such as ~48% for *E*. *coli* and *S*. *typhimurium*, and ~34% for *M*. *tuberculosis* and *P*. *aeruginosa*. We have previously reported that the fraction of bound sequence from ChIP-on-chip data begins to increase when the AT-content reaches ∼50% for H-NS from *S*. *typhimurium* and ∼38% for Lsr2 from *M*. *tuberculosis*, which correspond to the mean AT-content of the corresponding genome, respectively [29]. More interestingly, H-NS from *S*. *typhimurium* and Lsr2 from *M*. *tuberculosis* exhibit nearly identical binding patterns when the AT-content of the bound sequence is normalized against the mean AT-content of the respective genome. We have suggested that the “RGR” AT-hook-like motif employed primarily in xenogeneic silencers from *M*. *tuberculosis* (Lsr2) and *B*. *vietnamiensis* (Bv3F), both with low AT-content genomes, enables tighter binding to sequences of relatively lower AT-content [29]. In comparison, the “QGR” AT-hook-like motif in H-NS from *S*. *typhimurium* and *E*. *coli*, both with high AT-content genomes, has lower affinity towards mildly AT-rich DNA. Unexpectedly, the DNA binding affinity of the DNA binding domain of MvaT from *P*. *aeruginosa* is lower than that of Lsr2 from *M*. *tuberculosis*, both with similar low AT-content genomes, while it is similar to that of H-NS from *S*. *typhimurium* with high AT-content genome. Our biochemical and structural studies revealed that MvaT prefer flexible DNA sequences with multiple TpA steps and can better tolerate GC insertion in AT-rich sequences. It may follow that *Pseudomonas* has evolved MvaT with considerable GC-tolerance to cope with its low AT-content genome. It may also be possible that specific features like TpA steps are underrepresented in the *Pseudomonas* “core” genome compared to the mobile genome. It is still not clear why MvaT from *Pseudomonas* employ a different solution to distinguish foreign from self DNA, and the determinants may lie in detailed sequence characteristics of its genome.

While previous studies have been mainly focused on revealing functional similarities of xenogeneic silencers from different bacteria, their differences are largely overlooked. It is apparent that the abilities of xenogeneic silencers from different bacteria to distinguish foreign from self DNAs should be dependent on the mean AT-content of their corresponding genomes, and it is even possible that these xenogeneic silencers have to fine-tune their DNA binding properties to cope with different sequence characteristics of their own genomes and foreign DNAs. More studies should be inspired to further explore the correlation between the molecular mechanisms of xenogeneic silencers from different bacteria and the characteristics of their genomes.

## Materials and Methods

### Bacterial strains and chemicals

*P*. *aeruginosa* strain PAO1 Δ*mvaT cupA lacZ* has been described previously [23]. *E*. *coli* DH5αF’IQ (Invitrogen) was used as the recipient strain for all plasmid constructions. *E*. *coli* strain BL21 (DE3) was used for protein purification.

When growing *P*. *aeruginosa*, gentamicin was used at 25 μg/ml for liquid cultures and 30 μg/ml for solid media. Phase-ON and phase-OFF colonies of the reporter strain were visualized following growth on LB agar containing 75 μg/ml X-Gal.

### NMR sample preparation

DNA sequence encoding the C-terminal domain of MvaT (residues 77–124) from *P*. *aeruginosa* was subcloned into the pET21b vector, directly upstream of the His_6_-tag coding sequence. Point mutations were generated using the site-directed mutagenesis kit (SBS Genetech). *E*. *coli* BL21 (DE3) strain harboring the plasmid was cultured in LB medium at 35°C, and protein was over-expressed by induction with 100 μM IPTG until OD_600_ > 1.0. For ^15^N and ^13^C isotopically labeling, the bacteria were first grown in LB medium till OD_600_ > 0.9, then collected and resuspended in ^15^N, ^13^C-labeled M9 minimal medium for continuing growth, and 100 μM IPTG was added to induce protein expression after 40 min. The Cells were harvested by centrifugation 9 h after induction and resuspended in lysis buffer (50 mM Tris-HCl, 1 M NaCl, 20 mM imidazole, pH 9.0), then lysed by freezing and thawing, followed by sonification. After centrifugation, the supernatant containing the His-tagged fusion protein was applied to Ni-NTA affinity column (Qiagen) and eluted by elution buffer (50 mM Tris-HCl, 1 M NaCl, 250 mM imidazole, pH 9.0). Protein was further purified with size exclusion chromatography on a superdex 75 column (Amersham) in 50 mM sodium phosphate with 50 mM NaCl (pH 6.0). DNA samples were prepared by hybridization of self-complementary oligonucleotides, first heated to 94°C for 5 min and then annealed by slowly cooling down to room temperature. Protein/DNA complex samples were prepared by mixing the protein and DNA duplex at a 1:1 ratio.

### NMR resonance assignments

The NMR sample of free MvaT_ctd_ contained ~1 mM uniformly ^15^N, ^13^C—labeled protein in 50 mM sodium phosphate, 50 mM NaCl (pH 6.0) with 90% H_2_O/10% D_2_O, along with 0.01% NaN_3_ and 0.01% DSS. The NMR sample of MvaT_ctd_/DNA complex contained ~1 mM 1:1 complex of uniformly ^15^N, ^13^C—labeled protein and unlabeled DNA in the same buffer. All NMR spectra were collected at 298 K on 600, 700 or 800 MHz Bruker Avance spectrometers equipped with triple-resonance cryoprobes. Proton chemical shifts were referenced directly to DSS. ^15^N and ^13^C chemical shifts were referenced indirectly to DSS. Backbone and aliphatic side chain resonances were assigned by 2D ^1^H-^15^N HSQC, 2D ^1^H-^13^C HSQC, 3D HNCACB, 3D CBCA(CO)NH, 3D HNCO, 3D HBHA(CBCA)(CO)NH, 3D (H)CCH-COSY, 3D (H)CCH-TOCSY and 3D H(C)CH-TOCSY. Aromatic resonances were assigned using 3D ^1^H-^13^C-edited-NOESY optimized for aromatic resonances. Resonances of DNA complexed with MvaT_ctd_ were assigned using 2D F1, F2-^15^N/^13^C-filtered NOESY spectrum. There is a single set of resonances for the two strands of the palindromic 3AT duplex for the MvaT_ctd_/DNA complex sample, indicating that the binding process is fast on NMR time scale. Free DNA resonances were assigned with 2D ^1^H TOCSY and 2D ^1^H NOESY spectra. Data were processed using NMRPipe [65] and analyzed by NMR View [66].

### NMR titration experiments

The NMR samples used for DNA titration experiment all contained 0.1 mM uniformly ^15^N labeled protein in 50 mM sodium phosphate, 50 mM NaCl (pH 6.0) with 90% H_2_O/10% D_2_O. A series of 2D ^1^H-^15^N HSQC spectra with gradually increased DNA concentration (0.02 mM, 0.04 mM, 0.08 mM, 0.12 mM, 0.16 mM and 0.2 mM) were collected at 298 K on a Bruker Avance 500 MHz spectrometer with a triple-resonance cryoprobe and the chemical shifts changes were analyzed.

2D ^1^H NOESY experiments were performed to investigate the chemical shift perturbations for the DNA duplex d(CGCATATATGCG)^2^ samples with or without MvaT_ctd_ on a Bruker Avance 800 MHz spectrometer equipped with cryoprobe at 298 K. The NMR sample contained 0.4 mM DNA in 50 mM sodium phosphate, 50 mM NaCl (pH 6.0) with 100% D_2_O, and lyophilized protein powder of MvaT_ctd_ was added to final concentrations of 0.25 mM and 0.5 mM. The fingerprint region of intraresidual H1’-H6/H8 NOE cross peaks was analyzed.

For netropsin competition experiment, 0.1 mM uniformly ^15^N labeled MvaT_ctd_ was mixed with 0.2 mM DNA duplex in 50 mM sodium phosphate, 50 mM NaCl (pH 6.0) with 90% H_2_O/10% D_2_O. 2D ^1^H-^15^N HSQC spectra were collected with gradual addition of netropsin at concentrations of 0.05 mM, 0.1 mM, 0.2 mM, 0.3 mM and 0.5 mM, on a Bruker Avance 500 MHz spectrometer with a triple-resonance cryoprobe.

### Structure determination

Distance restraints were derived from 3D ^1^H-^15^N-edited NOESY-HSQC and 3D ^1^H-^13^C-edited NOESY-HSQC experimental data. Intermolecular NOEs were obtained from 3D F1-^15^N/^13^C-filtered, F2-^13^C-edited NOESY and 2D F1-^15^N/^13^C-filtered, F2-^15^N-edited NOESY spectra. Additional intermolecular NOEs were obtained by analyzing the 3D ^1^H-^15^N-edited NOESY-HSQC and 3D ^1^H-^13^C-edited NOESY-HSQC spectra of MvaT_ctd_/DNA complex. Protein dihedral angle restraints were obtained using TALOS+ [67]. Restraints on side chain χ^1^ angles were derived based on the intra-residual NOEs patterns.

For the structure calculation of free MvaT_ctd_, initial structures were generated by CYANA with restraints from the CANDID module [68]. The initial structures were then used as filter models to refine the NOE assignments and distance restraints using SANE [69]. These refined distance restraints were then used to calculate the refined structures with DYANA module of CYANA. This procedure was carried out iteratively as the refined structures can be used as the filter models for next round of SANE-DYANA calculation. When there was no distance violation larger than 0.5 Å, 100 structures with the lowest target function values from the 200 structures calculated with DYANA were selected for further refinement using AMBER 12 [70] using the generalized Born (GB) solvation model [71]. SANE was also used for the refinement process until there was basically no distance violation bigger than 0.2 Å and no angle violation was bigger than 5°. The top 20 structures with the lowest AMBER energies were selected for representation, and a mean structure was generated using SUPPOSE and energy minimized by AMBER 12. The quality of structures was analyzed using PROCHECK-NMR [72].

For the complex structure calculation, structures of MvaT_ctd_ and DNA were first calculated and refined separately, as described above. In addition to the NOE distance restraints, theoretical restraints for B-form DNA were also used in defining the structure of DNA. The α, β, γ, δ, ε and ζ backbone torsion angles of DNA were restrained to ranges -60° ± 30°, 180° ± 30°, 60° ± 35°, 130° ± 50°, 225° ± 75° and -95° ± 35° (or 180° ± 30°), respectively. Sugar pucker was fixed to C2’-endo by setting the pseudorotation phase angle P to a range of 135° ± 45° as implemented in AMBER program. Glycosidic torsion angle χ were set to anti conformation with a value of -120° ± 40°. Hydrogen bonds were used to maintain the typical Watson-Crick base-pairing. The protein/DNA complex structure was obtained and further refined in AMBER 12 using the GB solvation model by combining the MvaT_ctd_ and DNA structures with intermolecular NOE restraints. Briefly, coordinates of protein and DNA were arbitrarily combined, and the complex structure was calculated with the addition of intermolecular distance restraints all set to 50 Å, and gradually decreased to 20 Å, and 8 Å, then to the actual restraint distance, while the weight for intermolecular distance restraints was increased from 0 to 2 to 20 to 25 kcal/mol · Å^2^. The top 20 structures with lowest AMBER energies from the calculated 100 structures were selected and analyzed using PROCHECK-NMR. The mean structure was generated using SUPPOSE and energy minimized by AMBER 12. DNA helical and groove parameters were analyzed using the program CURVES+ [73].

### Electrophoretic mobility shift assay

The *cupA1* 340 bp fragment was PCR amplified from *P*. *aeruginosa* PAO1 genomic DNA using primers GT044 (5’ GCGAAGCCGTGGTTCGAGTTGTT) and GT045 (5’ ATCCCGGCCTCTCTTGCTTGTCTT). A 204 bp fragment of the *PA3900* gene was PCR amplified from the same genomic DNA using primers GT049 (CCGCAGGTGGCTGAACA) and GT050 (CGAATGCGGTGCGTTGATGG). PCR products were 5' end-radiolabeled with γ-^32^P ATP using T4 polynucleotide kinase (New England Biolabs). 400 nM DNA fragment, 4 μL γ-^32^P ATP (3,000 Ci/mmol, 10 mCi/mL, Perkin Elmer), 1x polynucleotide kinase buffer and T4 polynucleotide kinase enzyme (New England Biolabs) were incubated in a total of 40 μL at 37°C for 30 min. The reaction was stopped by the addition of 1 μL of 0.5 M EDTA and excess radioisotopes were removed using a G-25 spin column (GE Healthcare Life Sciences). Spin column resin was resuspended by vortexing the column upside down for 30 sec. The cap was loosened one-quarter turn and the bottom closure was snapped off. The column was then placed into a 1.5 mL microcentrifuge tube and spun at 2,800 rpm (735xg) in a tabletop centrifuge. The column was then placed into a fresh 1.5 mL microcentrifuge tube and the radiolabeling reaction was applied. DNA was eluted by spinning at 2,800 rpm (735xg) for 2 min in a tabletop centrifuge. 760 μL H_2_O was added to a final volume of 800 μL to dilute the DNA to a working stock of 20 nM. DNA was aliquoted and stored at -20°C. 1 μL of this stock will yield a final concentration of 1 nM in a 20 μL EMSA binding reaction. 1 nM radiolabeled DNA was incubated with varying concentrations of protein in binding buffer (15 mM HEPES pH 7.9, 40 mM KCl, 1 mM EDTA, 0.5 mM DTT, 5% glycerol). Addition of varying amounts of protein altered the concentrations of solutes from tube to tube. Buffer conditions were therefore normalized such that each reaction contained 8 mM Tris pH 8.0, 15 mM HEPES pH 7.9, 40 mM NaCl, 40 mM KCl, 1.4 mM EDTA, 0.5 mM DTT, 9.95% glycerol. Binding reactions were incubated at room temperature for 15 min before the addition of excess cold (unlabelled) DNA, where appropriate. The samples were further incubated another 15 min at room temperature. 2.5 μL of 10x DNA loading dye (10 mM Tris-HCl pH 7.5, 10 mM EDTA, 65% sucrose, 0.3% bromophenol blue) was added to each reaction and samples were loaded onto a 6% native polyacrylamide gel that had been pre-run for 1 h at 100 volts at 4°C. Samples were run at 70 volts for 165 min at 4°C, dried in a Gel Dryer (Labnet International) for 1 h at 80°C and exposed overnight on a storage phosphor screen (GE Healthcare Life Sciences/Molecular Dynamics). Gels were visualized the following morning using a Typhoon 9400 imager with an image resolution of 50 μm. The apparent dissociation constant, which corresponds to the protein concentration when the DNA is half bound, was determined by curve fitting based on quantified intensities of unbound DNA bands. The average of four replicates and their standard errors is reported.

### Protein binding microarray

PBM experiments were performed as previously described [29]. Sequence encoding full length MvaT from *P*. *aeruginosa* was cloned into vector pTH6838 [74] using the isothermal assembly method [75] to generate a vector producing a chimeric protein with glutathione S-transferase (GST) attached to the N-terminus of MvaT. Data was parsed with a custom Python script (written by and available from W.W.N) and plotted using the ggplot2 data visualization package [76].

### Isothermal titration calorimetry

ITC experiment was performed using MicroCal iTC200 system (GE Healthcare) at 283 K. 0.15 mM DNA was placed in the cell and titrated with MvaT_ctd_ or its mutants (2.3 mM–4.2 mM) injected in 2.4 μL aliquots. Corresponding “protein to buffer” controls were performed for background correction. ITC titration data were analyzed using Origin 7.0 (OriginLab) provided with the instrument. Standard deviation was calculated by according to the fit by Origin.

### β-Galactosidase assays

Cells of *P*. *aeruginosa* were permeabilized with sodium dodecyl sulphate and CHCl_3_ and assayed for β-galactosidase activity as described previously [77]. Assays were performed twice in triplicate on separate occasions. A representative data set is shown.

### MvaT-V mutant expression vectors

Plasmid pPSV-MvaT-V, that allows expression of the WT MvaT with a C-terminal vesicular stomatitis virus-glycoprotein epitope-tag (MvaT-V), has been described previously [30]. Site-directed mutagenesis of *mvaT* was carried out by the PCR to introduce mutations specifying amino acid substitutions R80A, K81A, K83A, K97A, N100A, K102A, K105A and K108A. Mutant coding sequences were digested with *Bam*HI and *Xho*I and cloned into pPSV-MvaT-V cut with the same enzymes, to generate plasmids pPSV-MvaT(R80A)-V, pPSV-MvaT(K81A)-V, pPSV-MvaT(K83A)-V, pPSV-MvaT(K97A)-V, pPSV-MvaT(N100A)-V, pPSV-MvaT(K102A)-V, pPSV-MvaT(K105A)-V and pPSV-MvaT(K108A)-V.

### Western blot

Production of the MvaT-V proteins was confirmed by Western blotting using an anti-VSV-G antibody (Sigma-Aldrich). An antibody against the α-subunit of RNA polymerase (Neoclone) was used to control for differences in protein loading.

## Supporting Information

S1 Fig Structural parameters predicted by DNAshape for MvaT-bound 100% AT 8-mer sequences with highest 20 (left panel) and lowest 20 (right panel) *E*-scores. Helix parameters are not calculated for the 2 bases at either end of the 8-mer sequence.
(TIF)

S2 Fig Resonance assignments for MvaT_ctd_ and the 3AT dodecamer in free and bound form. 2D ^1^H-^15^N HSQC spectra of free (A) and DNA-bound MvaT_ctd_ (B). Assignments are indicated by one-letter amino acid code and the sequence number. Finger print region of 2D ^1^H NOESY spectra of free (C) and protein-bound (D) 3AT DNA duplex. Intraresidue H1’-H6/H8 NOE peaks are labeled by base type and number, and sequential connectivity is shown by lines.
(TIF)

S3 Fig Effects of single mutation on MvaT_ctd_ structure. An overlay of 2D ^1^H-^15^N HSQC spectra of R80A (A), K81A (B), K83A (C), K97A (D), N100A (E), K102A (F), K105A (G) and K108A (H) (red) with that of WT MvaT_ctd_ (black). For K83A, the NH signals are not well dispersed indicating that protein fold of K83A may be changed. For other mutants, most of the affected NH signals are from residues close to the mutation site. Residues displaying significant chemical shift changes are indicated by one-letter amino acid code and residue number.
(TIF)

S4 Fig Effects of single mutation on DNA binding of MvaT_ctd_. Overlay of 2D ^1^H-^15^N HSQC spectra with different DNA/protein ratios for WT MvaT_ctd_ (A), R80A (B), K81A (C), K97A (D), N100A (E), K102A (F), K105A (G), and K108A (H). The DNA to protein ratios are 0 (black), 0.2 (red), 0.4 (green), 0.8 (blue), 1.2 (yellow), 1.6 (magenta) and 2.0 (cyan).
(TIF)

S5 Fig ITC curve fitting for WT MvaT_ctd_ and its mutants. Binding isotherms of the calorimetric titration of WT MvaT_ctd_ (A), R80A (B), K81A (C), K97A (D), N100A (E), K102A (F), K105A (G) and K108A (H) to AT-rich DNA. (I) Binding isotherm of the calorimetric titration of WT MvaT_ctd_ to GC-rich DNA. The thermodynamic parameters derived from these titration experiments are shown (inset).
(TIF)

S1 Dataset Protein binding microarray data for MvaT. (XLSX)
