# Supplementary figures and images for: A Novel AT-Rich DNA Recognition Mechanism for Bacterial Xenogeneic Silencer MvaT
Source: PLoS Pathog. 2015 Jun 11;11(6):e1004967. doi: 10.1371/journal.ppat.1004967 (PMC4466236; doi:10.1371/journal.ppat.1004967)

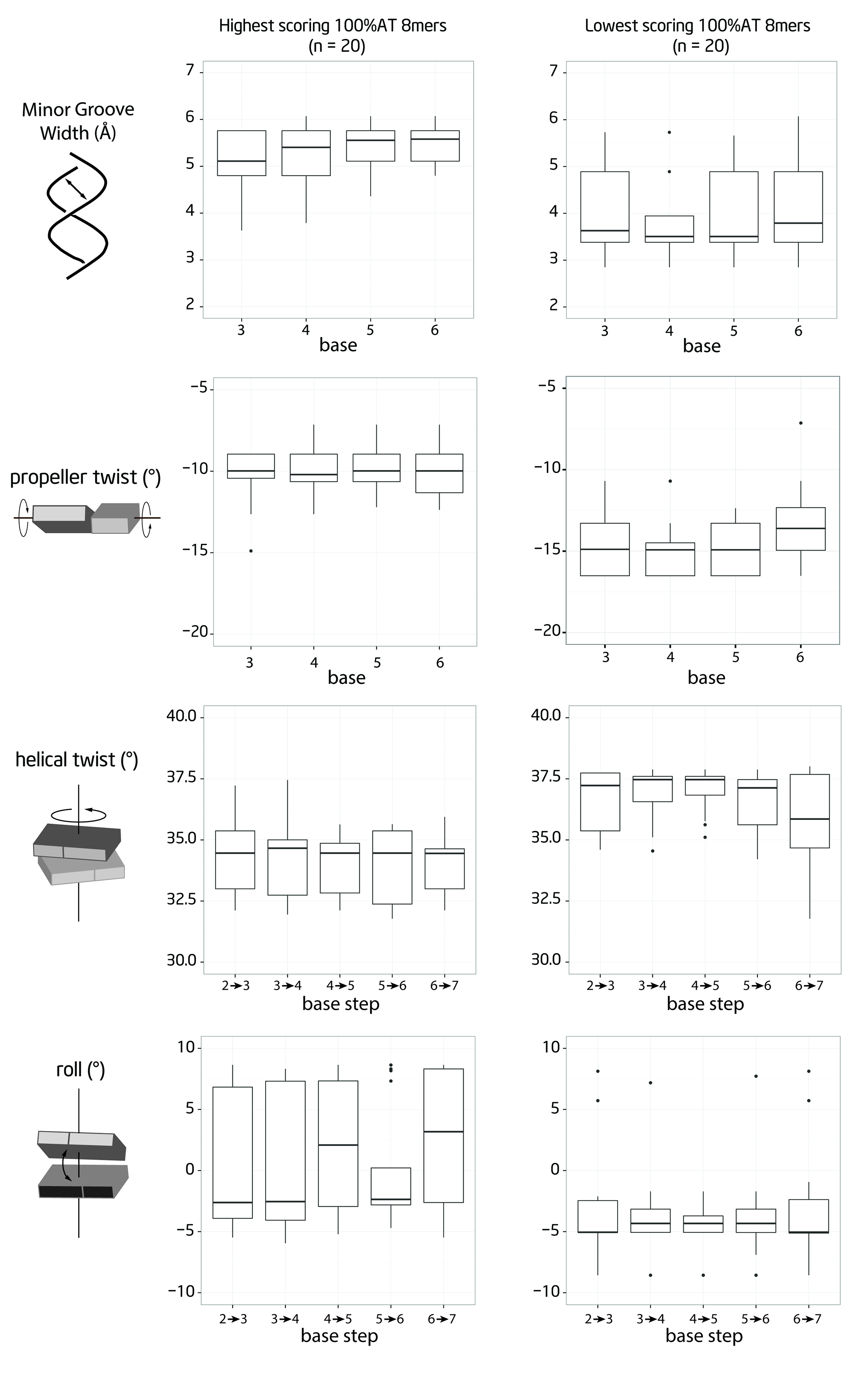

Supplement: S1 Fig — Helix parameters are not calculated for the 2 bases at either end of the 8-mer sequence. (TIF) [file ppat.1004967.s001.tif]

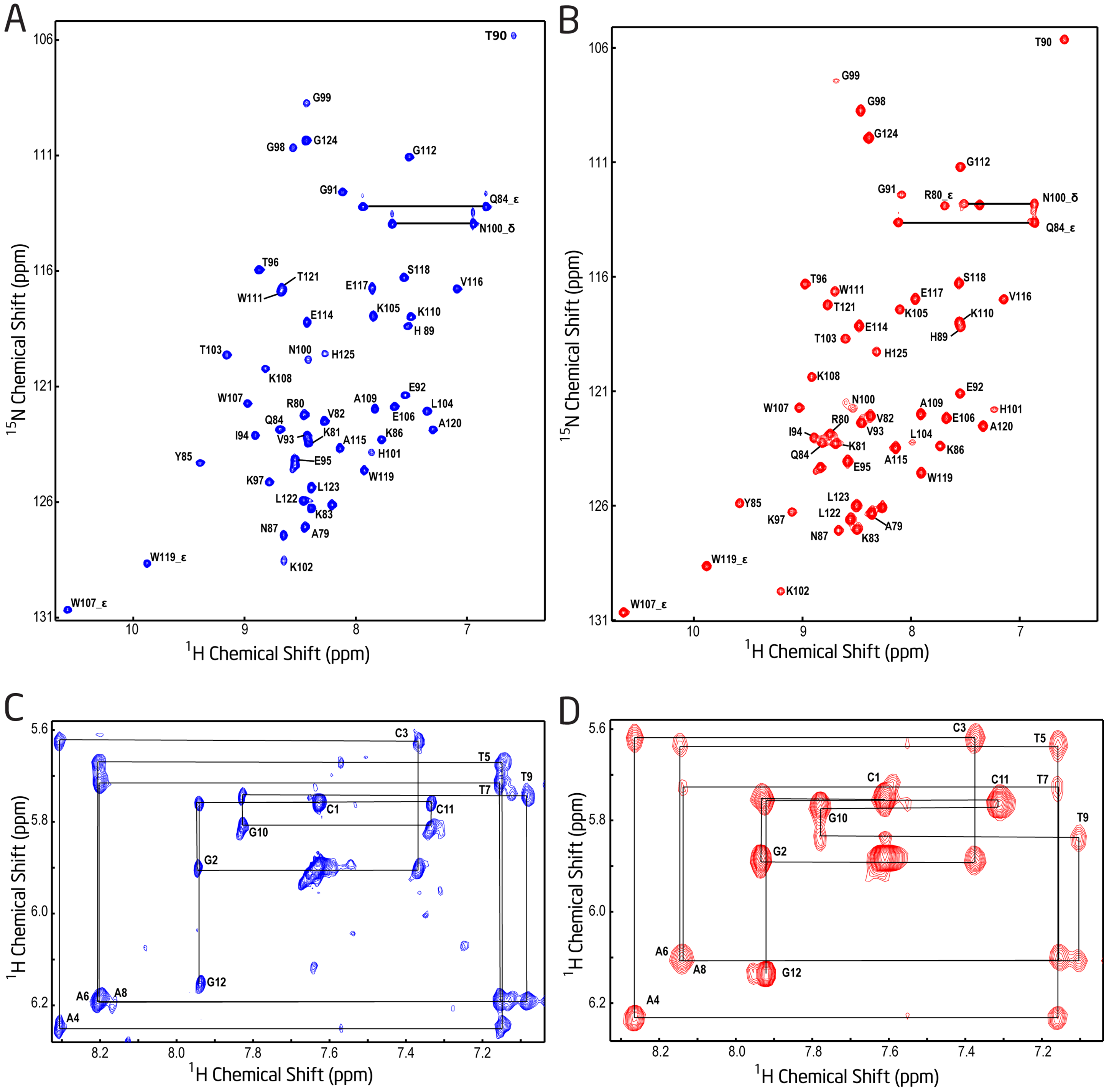

Supplement: S2 Fig — 2D 1H-15N HSQC spectra of free (A) and DNA-bound MvaTctd (B). Assignments are indicated by one-letter amino acid code and the sequence number. Finger print region of 2D 1H NOESY spectra of free (C) and protein-bound (D) 3AT DNA duplex. Intraresidue H1’-H6/H8 NOE peaks are labeled by base type and number, and sequential connectivity is shown by lines. (TIF) [file ppat.1004967.s002.tif]

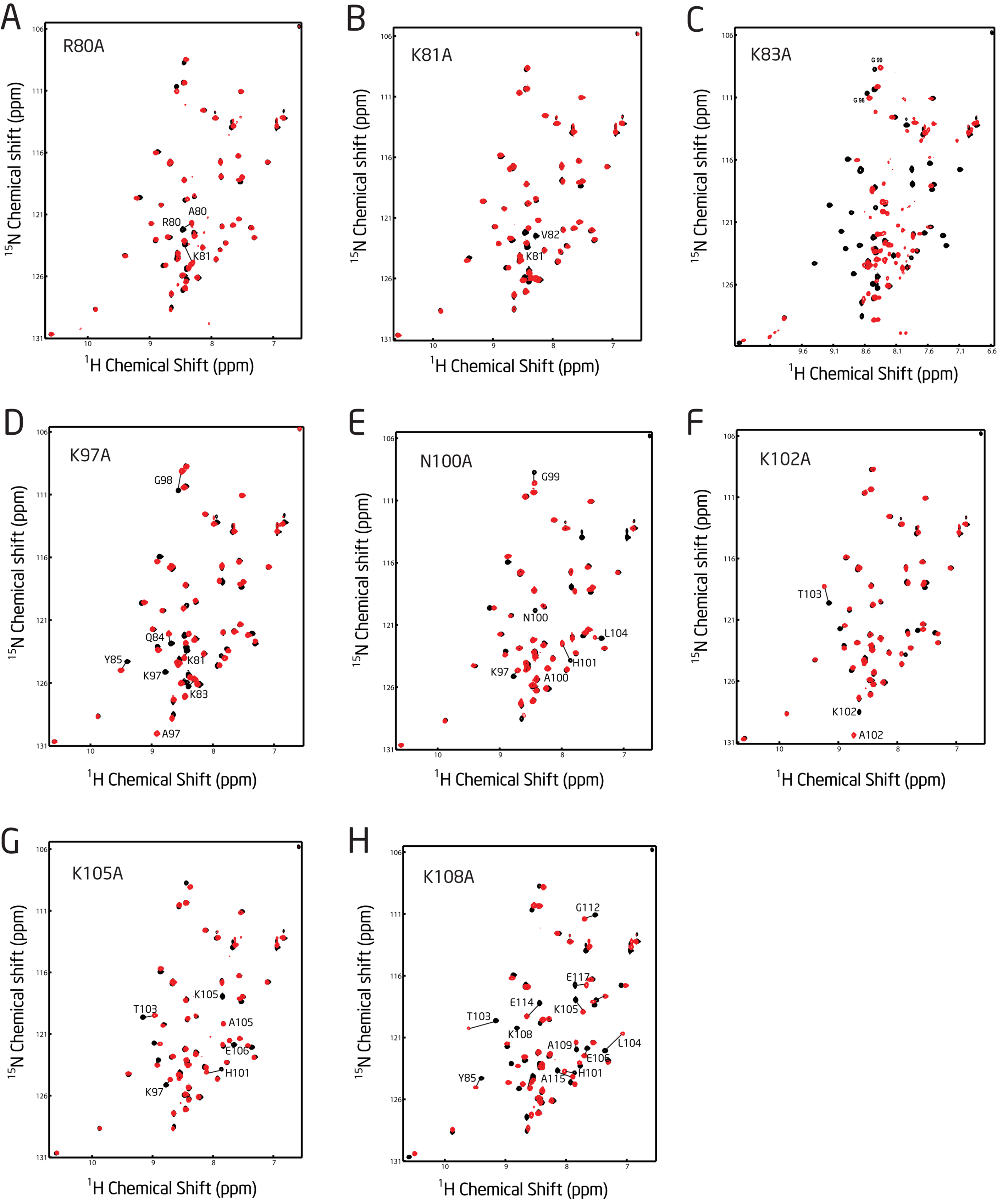

Supplement: S3 Fig — An overlay of 2D 1H-15N HSQC spectra of R80A (A), K81A (B), K83A (C), K97A (D), N100A (E), K102A (F), K105A (G) and K108A (H) (red) with that of WT MvaTctd (black). For K83A, the NH signals are not well dispersed indicating that protein fold of K83A may be changed. For other mutants, most of the affected NH signals are from residues close to the mutation site. Residues displaying significant chemical shift changes are indicated by one-letter amino acid code and residue number. (TIF) [file ppat.1004967.s003.tif]

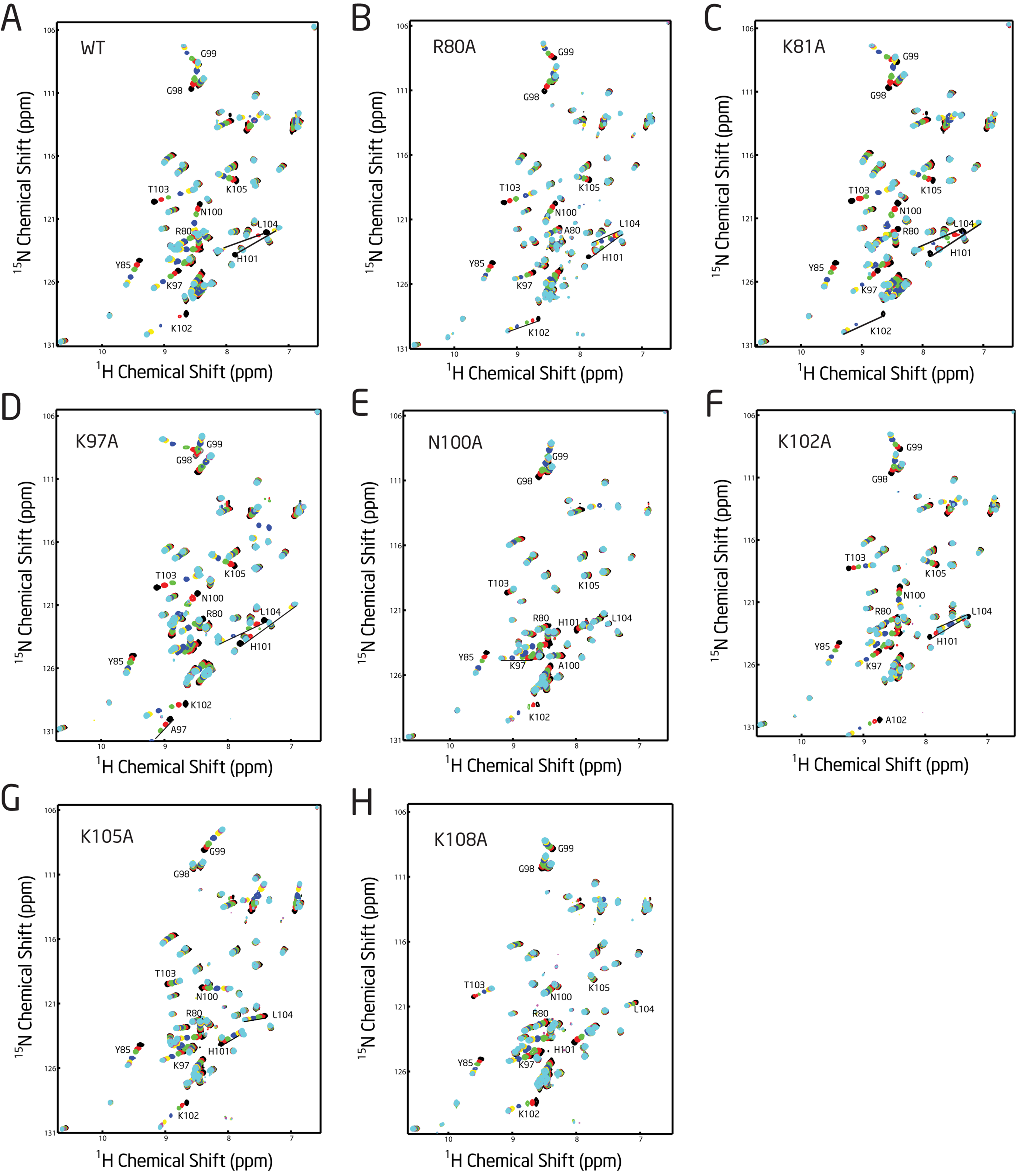

Supplement: S4 Fig — Overlay of 2D 1H-15N HSQC spectra with different DNA/protein ratios for WT MvaTctd (A), R80A (B), K81A (C), K97A (D), N100A (E), K102A (F), K105A (G), and K108A (H). The DNA to protein ratios are 0 (black), 0.2 (red), 0.4 (green), 0.8 (blue), 1.2 (yellow), 1.6 (magenta) and 2.0 (cyan). (TIF) [file ppat.1004967.s004.tif]

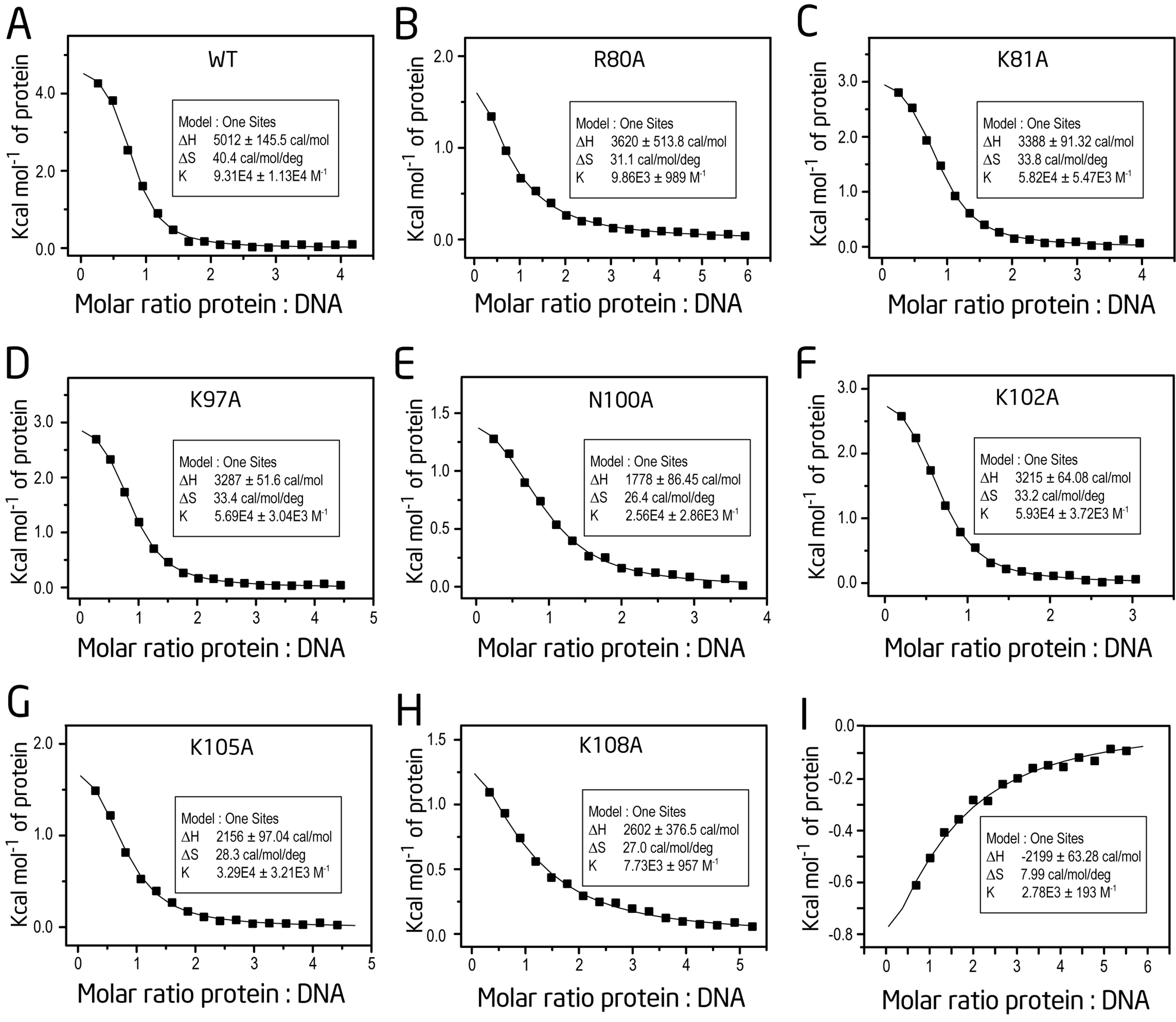

Supplement: S5 Fig — Binding isotherms of the calorimetric titration of WT MvaTctd (A), R80A (B), K81A (C), K97A (D), N100A (E), K102A (F), K105A (G) and K108A (H) to AT-rich DNA. (I) Binding isotherm of the calorimetric titration of WT MvaTctd to GC-rich DNA. The thermodynamic parameters derived from these titration experiments are shown (inset). (TIF) [file ppat.1004967.s005.tif]
